# Sex modulates the long-term effects of delivery mode on microbiota–gut barrier crosstalk and colitis susceptibility in mice

**DOI:** 10.1080/19490976.2026.2658276

**Published:** 2026-04-27

**Authors:** Bruna Maitan Santos, Jordi Estellé, Yuliaxis Ramayo-Caldas, Sead Chadi, Monica Barone, Florian Chain, Camille Kropp, Patrizia Bridigi, Philippe Langella, Rebeca Martín

**Affiliations:** a Paris-Saclay University, INRAE, AgroParisTech, Micalis Institute, Jouy-en-Josas, France; b Paris-Saclay University, INRAE, AgroParisTech, GABI, Jouy-en-Josas, France; c Animal Breeding and Genetics Program, Institute for Research and Technology in Food and Agriculture (IRTA), Torre Marimon, Caldes de Montbui, Spain; d Department of Pharmacy and Biotechnology, University of Bologna, Bologna, Italy

**Keywords:** Early-life, vaginal delivery, C-section, sex differences, colitis, microbiota

## Abstract

Sexual dimorphism and mode of delivery are key determinants of gut physiology and microbiota development and may differentially affect predisposition to gut-related diseases. Cesarean section delivery markedly shapes early-life microbiota, predisposing individuals to higher risk of immune and metabolic comorbidities later in life. Although both sex and delivery mode are known to influence gut barrier–microbiota crosstalk, whether delivery mode modulates or counter-regulates sex-specific features of this interaction remains, to our knowledge, largely unexplored. Here, we investigated how sex impacts gut barrier–microbiota crosstalk shaped by delivery mode across development until adulthood by reanalyzing existing data. Using a preclinical mouse model, we combined gut barrier analyses with differential abundance and co-occurrence network approaches (LinDA and NetMoss). We found that the impact of CSD on gut barrier–microbiota crosstalk is partially dependent on sex and life stage. During the first days of life, delivery mode dictates immune imprinting and microbial network topology, with only limited sex effects. However, trajectories diverged with age, with CSD males exhibiting colitis reoccurrence in adulthood. By applying integrative strategies to stratify data by sex and development, our study uncovers short- and long-term sex-dependent gut barrier and microbial signatures. These findings reveal that delivery mode might program sex-specific host-microbiota trajectories with consequences for gut health and disease susceptibility, highlighting the need to consider sex and early-life microbial imprinting in future microbiome-targeted interventions.

## Introduction

Biological sex refers to the intrinsic characteristic that distinguishes males from females. Sex influences the prevalence, course and severity of the majority of common diseases and disorders, including noncommunicable diseases.^1^^-^^5^ Sexual dimorphism has been explained by differences in chromosome genes, sex hormones and social environmental behaviours (drinking, smoking, etc.), although these factors do not completely explain the differences found.^2^ The gut microbiota has been found to have a significant impact on the pathogenesis of several chronic diseases. Interestingly, the composition of the gut microbiota also varies between sexes.^2^ Numerous studies in both mice and humans have found sex differences in beta-diversity and alpha-diversity in the gut microbiota^6^^,^^7^ and sex have been found among the ten major factors that explain taxonomic human gut microbiota variations.^8^ Nevertheless, a consistent pattern of community-level sex differentiation has not been found, and the proposal of robust and reliable microbial indicators of sex has not been made possible due todifferences among studies.^9^ Overall, while sex can contribute to gut microbiome development, the complexity of factors that interact and influence its development impedes consistency across studies to confirm sex effects and characterize them. As a proof, only one human study has described that infant sex as a dominant contributor to infant gut microbial development.^10^

C-section delivery (CSD) is one of the most common surgeries in the world, accounting for approximately 21% of all childbirths worldwide and being more than 40% of births in some countries, such as Brazil.^11^^-^^14^ In 1985, the World Health Organization (WHO) recommended that CSD rates should be lower than 10%–15%.^14^ However, CSD rates have continued to rise, mainly owing to personal or medical convenience,^11^^-^^13^ and projections suggest that by 2030, the global CSD rate will be nearly 30%.^15^ CSD, which is mainly elective (without labor), has been related to an increased risk of noncommunicable diseases (NCDs). Several meta-analyses have shown that infants born via CSD are at a higher risk of developing allergies, asthma, IBD and obesity, among others.^16^^-^^29^ Since these children have an altered bacterial community, it has been hypothesized that these alterations lead to differences in mucosal tolerance and disease risk later in life.^30^^,^^31^

It is now recognized that sex bias exists in biomedical research, which is the basis of clinical research and medical decision making.^32^ Traditionally, female individuals have been underrepresented in research studies, both in randomized clinical trials and in preclinical animal trials.^32^^-^^34^ Although sex differences in murine models are well known, they are often mistreated by only analyzing one sex, most often males.^35^^-^^37^ Sex has been found to be a factor in the CSD association with NCDs. For instance, a meta-analysis has found that asthma has a higher incidence in CSD female offspring compared to VD ones while no differences have been found in males.^28^ Also, the CSD risk of obesity^38^^-^^40^ seems higher for boys than for girls in Canada and US cohorts,^41^^,^^42^ but the opposite in a Chinese cohort.^43^ Regarding neurodevelopmental disorders, CSD effects have been associated with an increased risk of neurodevelopmental disorders in males and with an increased risk of motor delay in females.^44^ Another important CSD effect characterized its association with inflammatory bowel diseases,^22^^-^^24^ with some studies showing potential sex effects.^45^^,^^46^ Taken together, understanding the sexual dimorphism in diseases is essential to investigate the pathogenesis of some chronic diseases and better deciphering the role of CSD in their increased incidence.

Here, our primary objective was to determine whether susceptibility to colitis reocurrence later in life differs according to sex in CSD animals. As secondary objectives, we investigated whether CSD alterations in gut barrier integrity, immune markers, and the microbiota centrality network during early life could be associated with sex differences in disease susceptibility later in life. Finally, we analyzed the microbial network structure and abundance across multiple developmental stages to evaluate potential patterns. It is important to highlight that this study was conducted by reanalysing pre-existing preclinical data,^47^ in which sex was not considered as a biological variable.

## Materials and methods

### Animals

All procedures were approved by the regional ethics committee (Comethea) and adhered to EU Directive 2010/63/EU on animal protection. Experiments were conducted under specific pathogen-free (SPF) conditions under controlled ambient temperature (21 °C) and humidity with water and food *ad libitum*. All in vivo experiments were conducted at the National Research Institute for Agriculture, Food and Environment (INRAE), within the Infectiology of Farm, Wildlife and Laboratory Animals Platform (IERP) in Jouy-en-Josas, France. Timed pregnant RjOrl:SWISS mice (Janvier Labs, Le Genest-Saint-Isle, France) were used.

### Mode of delivery model and experimental timeline

This study is based on previously published data comparing cesarean-delivered (CSD) and vaginally delivered (VD) animals,^47^ which were reanalyzed here with a focus on sex-dependent effects. On gestational day 19, dams RjOrl:SWISS (Janvier Labs; Le Genest-Saint-Isle, France) underwent hysterectomy; neonates were immediately fostered by surrogate dams that had delivered vaginally within the previous 12 h (C-section delivery, CSD). Control litters were obtained via spontaneous vaginal delivery (VD). To minimize potential litter effects, VD pups were cross-fostered among dams delivering in the same time window. Offspring of both sexes were group-housed and separated by sex after weaning.

**Figure 1.**
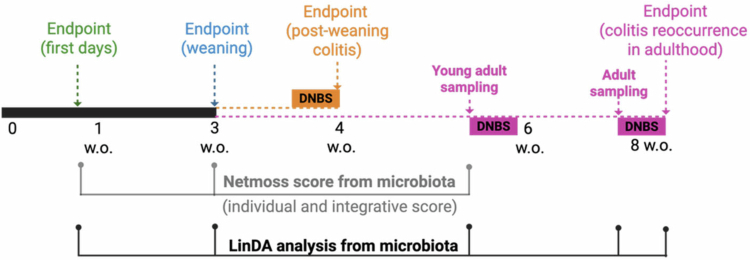
Experimental timeline of VD vs. CSD analyses across life stages to evaluate sex effects. Schematic representation of euthanasia and fecal sampling time points from birth to adulthood (~8 weeks old, w.o.), specifically, first days of life (1 w.o. in green), weaning (3 w.o., in blue), postweaning colitis (4 w.o., in orange), and colitis reoccurrence in adulthood (8 w.o. in pink) endpoints and 6 w.o. (young adult stage, in pink) and 4 d before completing 8 w.o. (in a period of colitis remission prior to DNBS rechallenge in pink) fecal sample collection. DNBS-induced colitis was performed at 4 w.o. mice to assess acute colitis susceptibility and at 6 and 8 w.o. to evaluate colitis reoccurrence. The microbiota datasets were analyzed using NetMoss (to compute individual and integrative network scores, with timepoints indicated by gray lines) and LinDA (to assess differential taxa abundance, with timepoints connected by a black line) at each time point, allowing the evaluation of delivery- and sex-dependent effects throughout early life and into adulthood. Created in BioRender (https://BioRender.com/ew680ex).

As illustrated in the experimental timeline (Figure 1), our analyses were performed in both female and male VD or CSD offspring from the first days of life (5 d, in green) until adulthood after colitis reoccurrence (8 weeks, in pink). To better picture each stage of life, graphs followed the same color scheme as the corresponding age in the timeline. At each life stage, the parameters analyzed for potential sex effects were those that showed an impact of CSD at the corresponding age.

### Colitis-induced models

Two models were used in this analysis. The acute colitis model involved 4-week-old mice that were anesthetized (0.1% ketamine + 0.06% xylazine, i.p.) and received 2,4-dinitrobenzene sulfonic acid (DNBS, 200 mg/kg, Sigma-Aldrich, France) intrarectally in 50 µL of 30% ethanol in sterile phosphate-based saline (EtOH) to induce colitis in young adulthood. Vehicle controls received EtOH only.

A colitis reoccurrence model was used to induce chronic colonic inflammation. Four-week-old mice were submitted to chronic administration of DNBS as previously described.^48^ Briefly, mice were challenged with a first dose of DNBS (200 mg/kg), the same as that used in the acute colitis model. After 21 d of recovery, the colitis was reactivated using DNBS at the same dose. The control mice received only EtOH.

**Figure 2.**
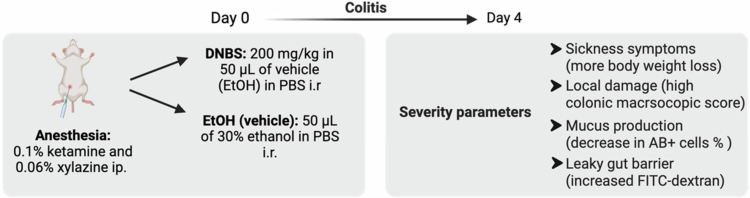
Brief visual explanation of colitis induction and the parameters used to measure its severity, such as the percentage (%) of body weight loss during DNBS-induced colitis to evaluate sickness symptoms, macroscopic assessment of colonic tissue to evaluate local damage, quantification of colonic goblet cells acid mucin (AB)+ to evaluate mucus production, and intestinal barrier integrity assessed by FITC-dextran translocation.

A brief explanation of the colitis induction and the parameters used to evaluate its severity can be found in Figure 2. Colonic macroscopic score measurements were conducted blindly.

### Quantification of serum lipocalin 2 and serum soluble CD14 (sCD14) levels

Serum lipocalin 2 levels were measured following kit instructions mouse lipocalin-2/NGAL duo Set ELISA (R&D systems, Inc., USA & Canada). Serum soluble CD14 (sCD14) levels were measured using sCD14 ELISA kit following the manufacturer's instructions (R&D systems, Inc., USA & Canada).

### Quantification of colonic myeloperoxidase (MPO) activity and colonic cytokines measurement

Colonic samples were thawed and homogenized in 0.5% hexadecyltrimethylammonium bromide (HTAB) prepared in 50 mM potassium phosphate buffer (pH 6.0) using a Precellys 24 homogenizer (Bertin Technologies, France) with ceramic beads (3 × 30 s at 6,000 rpm, 4 °C). The homogenates were centrifuged at 10,000 × g for 15  min at 4 °C, and the resulting supernatants were collected for MPO and cytokine measurements.

MPO activity was determined spectrophotometrically by mixing 10 µL of the homogenate with 200 µL of reaction buffer containing o-dianisidine dihydrochloride and hydrogen peroxide (H₂O₂). The change in absorbance was measured at 450 nm for 5 min at 25 °C using a microplate reader. MPO activity was expressed as units per gram of tissue (U/g) as previously described.^49^

Colonic IL-1α, IL-1β, IL-6, IL-10, IL-12p70, IL-17A, IL-23, IL-27, MCP-1, IFN-β, IFN-γ, TNF-α, and GM-CSF cytokines were determined with LEGENDplex™ mouse inflammation panel according to the manufacturer's instructions (Biolegend, USA).

### Histological analyses

Histological features were assessed using Alcian blue (AB) staining in accordance with standard protocols^50^^,^^51^ in the Histology facility of a Bridge platform of UMR 1313 GABI. Images were blindly analyzed with Panoramic Viewer software.

### Splenic T cells populations

Splenic cell suspensions from the first days of life at the pups' endpoint were obtained by mechanical extrusion through a 40-μm nylon cell strainer (BD, Switzerland). The cells were strained using 1 mL of Dulbecco's modified Eagle's medium (DMEM, Gibco, France) supplemented with 10% fetal bovine serum (FBS, Gibco, France). Erythrocytes were lysed using buffer Hybri-Max (Sigma-Aldrich, USA) following specifications. The cell pellets were resuspended in 1 × 10^6^ cells and blocked with anti-CD16/32 and stained with panels CD3+CD4+Tbet+and CD3+CD4+ROR-γ+ using eBioscience antibodies (France). The samples were subsequently analyzed using an Accuri C6 cytometer (BD). Data were acquired on an Accuri C6 flow cytometer (BD) and analyzed using CFlowSampler.

### Using chamber experiments

Segments of the ileum and colon were opened along the mesenteric border and mounted in Ussing chambers (P2300, 0.2 cm^2^ exposed area) with oxygenated Krebs buffer (glucose on the serosal side; mannitol on the mucosal side) at 37 °C. Transepithelial conductance and short-circuit current were recorded under a voltage clamp. To assess paracellular permeability, TRITC (4 kDa, TdB Labs, Sweden) was added to the mucosal chamber (0.4 mg/mL). Serosal samples were collected every 15 min for 2 h and analyzed enzymatically. Trans-epithelial conductance was measured by clamping the voltage and recording the change in the short-circuit current (Isc) Carbachol (100 µM) was added serosally at the end of the experiments to verify tissue viability.

### Microbial DNA extraction and 16S sequencing analysis

Microbial DNA extraction was performed with standard procedures as previously reported^47^ In brief, DNA was isolated from colon tissue (postnatal day 5), colonic content (endpoint), and fecal samples collected at intermediate time points and from maternal feces, skin, and vaginal swabs using the QIAGEN Mouse Stool Mini Kit (QIAGEN, Hilden, Germany) according to the manufacturer's instructions. The V3–V4 region of the 16S rRNA gene was amplified with the primers PCR1F_343 and PCR1R_784, and the Illumina MiSeq platform (Illumina, USA) was used to sequence. The raw sequence data were deposited in the Sequence Read Archive under Bioproject PRJNA876103 (accessions SAMN30638467–SAMN30638852).

Bioinformatic analysis of 16S sequencing data was performed using DADA2 v1.28.0 R package^52^ following the author's recommendations. In brief, we applied the standard DADA2 pipeline with default parameters for (1) quality filtering of the raw reads, (2) denoising DADA2 algorithm, (3) merging paired‑end reads, (4) removing chimeric sequences, and (5) quantifying amplicon sequence variants (ASVs) in each sample. The taxonomic classification of each ASV was performed with the DADA2 naive classifier using the SILVA v.138.1 database^52^ as the reference taxonomy. Finally, the abundance table at the genera level was constructed by using the tax_glom funtion in the phyloseq v1.46.0 R package.^53^

### NetMoss analysis

The resulting genus abundance table was used for diversity analyses and aggregated to the genus level for co-occurrence Sparcc network inferences between the VD and CSD groups with NetMoss.^54^ Network graphical representation was created with CytoScape,^38^ and the topological parameters of the network and “node” centrality values were calculated using the CentiScaPe plugin.^55^ Centrality measures, such as degree, quantify the importance of each bacterial genus within the network, reflecting its potential influence on microbial community structure and intermodule interactions. High-centrality nodes are interpreted as key taxa that may coordinate community dynamics, while low-centrality nodes represent more peripheral members. Modules within these networks were identified using the Weighted Gene Co-Expression Network Analysis. This step captures microbial taxa that cooperate within the same module while maintaining competitive interactions between modules. The contribution of each node (bacterial genus) to the transition between VD and CSD networks was calculated as follows:

$${\mathrm{NMSS}(i)}_{A\to B}=\sum_{j}^{\mathrm{Neighbor}s\;A}{∆D}_{\mathrm{ij}}-\sum_{l}^{\mathrm{Neighbors}\;B}∆D,$$

where *A* and *B* represent the VD and CSD networks, respectively. *D* denotes the differential module distance matrix, Neighbors *A* include all neighboring modules in the VD network, and Neighbors *B* represent all neighboring modules in the CSD network.

Microbial network approaches assume that co-occurrence patterns represent biologically meaningful interactions and that the resulting network topology reflects underlying dependencies among taxa. To ensure robustness, analyses were performed separately for females and males, and key driver taxa were identified using conservative NetMoss score thresholds (≥0.5 for weaning, ≥0.6 for early life). The intersection modules represent the stable elements during the transition, where the transition modules resulted in alterations in the network structure. In this way, the model quantifies the shift in module structure, highlighting microbial taxa that drive network changes between the experimental conditions. Nodes with high NetMoss scores correspond to genera that contribute most to network restructuring between VD and CSD conditions. Their centrality values provide additional biological insight: taxa with high centrality are likely influential in maintaining community stability or mediating interactions between modules, suggesting mechanistic roles in microbiota assembly. Receiver operating characteristic (ROC) curves and area under the curve (AUC) metrics were used to assess the discriminatory power of these taxa in classifying delivery mode.

### LinDA differential abundance analysis

Differential abundance analyses were performed using Linear Models for the differential abundance framework in the LinDA v0.2.0 R package.^56^ This method fits linear regressions on centered log-ratio (CLR) transformed abundance data, identifies a bias term owing to the compositional nature of microbiome data and corrects this bias using the mode of regression coefficients across taxa. The same raw reads were reprocessed using *p*-values derived from the bias-corrected coefficients and adjusted for multiple testing by Benjamini–Hochberg. The results are summarized as effect sizes and adjusted *p*-values. Differential abundance analyses were used in three models to test: (1) the effect of sex (male vs. female) corrected by delivery mode, (2) the effect of delivery mode (VD vs. CSD) corrected by sex, and (3) the interaction effect between sex and delivery mode (*i.e.*, a differential abundance model comparing four levels: VD_male vs. VD_female vs. CSD_male vs. CSD_female). In all three models, the effect of DNBS treatment was included as a cofactor for time points after the start of the first treatment. The addition of additional co-factors such as dams or adoptive dams was explored but not deemed necessary.

### Statistical analyses of mouse phenotypes

The strategy of this study to evaluate sex effects in VD and CSD animals was achieved by re-using pre-existing data^47^ with only parameters affected by CSD. The data distribution was evaluated using the Shapiro–Wilk test and Prism's distribution-likelihood analysis. Two- or three-way ANOVA was applied to evaluate the effects of delivery, sex, and DNBS analyses for normally distributed data with equal variances, followed by Fisher's test with multiple comparisons. When assumptions of normality or variance homogeneity were not met, the Kruskal–Wallis test was used, followed by Dunn's post hoc test with multiple comparisons. Both analyses were performed in Prism 11 (GraphPad Software, USA). The number of animals is referenced in each graph legend and detailed with attrition in Sup. Tables 1 and 2. *p*-values < 0.05 were considered statistically significant.

## Results

### Sex-dependent differences in colitis reoccurrence in CSD animals during adulthood

To determine whether susceptibility to colitis reoccurrence differs according to sex and delivery mode, we evaluated body weight loss as the primary endpoint of colitis severity, together with mucin production and inflammatory markers following DNBS rechallenge in adulthood.

**Figure 3.**
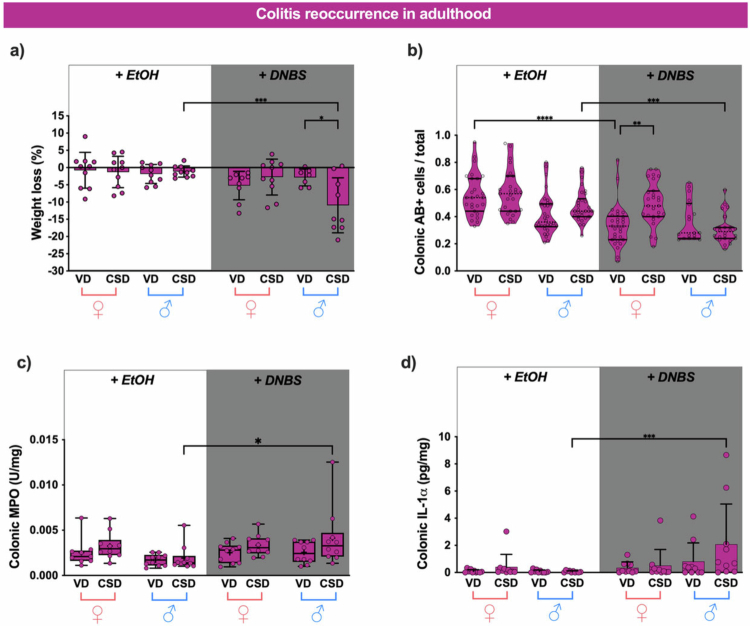
Cesarean delivery (CSD) exacerbates colitis reoccurrence severity in a sex-dependent manner in adult mice. (a) Percentage of body weight loss in colitis recurrence in adulthood. (b) Quantification of colonic goblet cells Ab +  after colitis reoccurrence in adulthood in technical triplicates or duplicates. (c) Colonic inflammatory marker myeloperoxidase (MPO) activity units (U) and interleukin (IL)-1α (d) concentrations per mg of tissue. The data are represented as scatter dot plots with individual animals for normal data (a, d). The mean values are indicated by black dotted lines, and the error bars denote the standard deviation (SD). Box plots and violin plots were used for non-normal distributed data (b and c). Statistical analyses were performed using Kruskal–Wallis tests followed by Dunn's post hoc test for non-normal data and three-way ANOVA for normal data, followed by Fischer's post hoc test. *N* = 10. * *p* < 0.05, **p < 0.01, ***p < 0.001, and ****p < 0.0001.

CSD males lost more weight compared to VD counterparts with colitis reoccurrence (Figure 3a). This effect was supported by a decrease in acid mucin (AB+) cells (Figure 3b), together with increased MPO activity (Figure 3c) and Il-1α levels (Figure 3d), reinforcing a stronger inflammatory response in CSD males after colitis reoccurrence than VD counterparts.

In contrast, females had no weight loss or decreased AB+ cells after colitis reoccurrence compared with their vehicle-treated counterparts (EtOH, Figure 3a and b, respectively). Instead, AB+ cells were increased in CSD females compared with those in VD females following colitis reoccurrence (Figure 3b). The lack of increase in MPO activity (Figure 3c) and Il-1α levels (Figure 3d) in all females with colitis reoccurrence supports the stronger impact of colitis reoccurrence in CSD males.

As previously described, CSD facilitates an exacerbated response to acute colitis in mice.^47^^,^^57^ As expected,^47^ DNBS caused marked body weight loss in all CSD groups (Supl. Figure 1). Another parameter used to evaluate the severity of the colitis induced by DNBS was local damage, which was measured using colonic macroscopic score,^47^ and male CSD animals showed a tendency toward a higher macroscopic score (*p* = 0.05) compared to VD counterparts, while no difference was detected between the female groups (Supl. Figure 1).

### First days' gut barrier immune development and gut microbial network centrality are shaped by delivery mode with low sex influence

To explore potential early-life factors that could precipitate colitis recurrence observed in CSD males during adulthood, we evaluated inflammatory and barrier-related parameters during the first days of life. Specifically, we profiled local inflammation (lipocalin 2, cytokines, and immune cells) and barrier integrity (sCD14) parameters in first days' pups born by VD or CSD. In parallel, the microbial network structure was analyzed to determine whether early microbial interactions differed according to delivery mode and sex.

**Figure 4.**
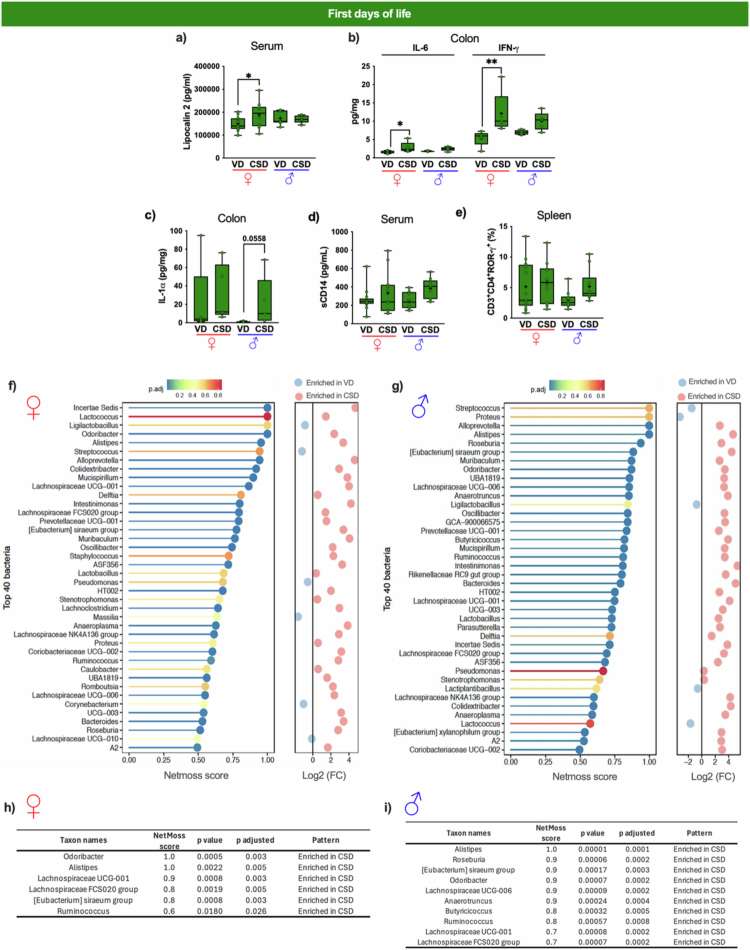
Immunological and gut microbiota alterations in first days of life influenced by delivery mode in females and males. (a) Serum lipocalin-2 concentrations (in pg/mL) in animals of both sexes (♀ females in red and ♂ males in dark blue) born by vaginal (VD) or c-section (CSD) delivery. (b) Colonic cytokines interleukin (IL)-6 and interferon (IFN)-*γ* (pg/mg). (c) Colonic IL-1α levels corrected by mg of protein (pg/mg). (d) Serum levels of soluble CD14 (sCD14, pg/mL). (e) Splenic CD3⁺CD4⁺RORγ⁺ T cell populations (%). (f–g) NetMoss scores showing top 40 microbial taxa with higher network centrality in CSD animals during the first days of life in females (f) and males (g). The fold change (FC) indicates the fold change in log2 abundance. (h-i) Tables showing butyrate-producing taxa with increased network centrality (NetMoss scores) in CSD females (h) and CSD males. (i) The color represents the endpoint in the experimental timeline. Statistical comparisons were performed using Kruskal–Wallis tests followed by Dunn's post hoc test. *p < 0.05, **p < 0.01. *N* = 10–15 animals per group.

Some inflammatory parameters that increased in CSD were significant only in CSD females (Figure 4a,b), specifically the increase in serum lipocalin-2 levels (Figure 4a) and colonic IL-6 and IFN-*γ* levels (Figure 4b), after pairwise comparisons. However, using linear modeling, CSD animals had increased lipocalin-2 levels [26,534 ± 10,762  pg/mL, *p* < 0.05; F(4, 55) = 97, *p* < 0.001], with only a trend for sex and delivery mode interaction (*p* = 0.07). Also, delivery mode had an impact on both cytokines [14.9 ± 5.6 pg/mL, *p* < 0.01; F(2, 37) = 6.40, *p* < 0.01 and 5.2 ± 1.9 pg/mL, *p* < 0.01; F(2, 37) = 8.11, *p* < 0.001, respectively], but again, there was no sex effect. CSD males had a potential impact on colonic IL-1α (Figure 2e, p = 0.05) after pairwise comparisons. IL-1α is usually released due to cellular damage caused by bacterial translocation, and CSD is associated with higher colonic IL-1α levels [14.9 ± 5.6 pg/mg, *p* < 0.01; F(8,110) = 7.58, *p* < 0.001] and male animals with low levels (–15.1 ± 5.6 pg/mg, *p* < 0.01; F(8,110) = 7.58, *p* < 0.001), but there is no delivery‒sex interaction. Even though they were impacted by CSD model during the first days of life,^47^ no alterations in serum soluble CD14 (sCD14; Figure 2c), an important marker of barrier disruption, or in the proportion of splenic CD3+CD4+ROR-γ+ Th17 cells (Figure 4d) were observed when groups were divided by sex after pairwise comparisons, showcasing the low effect of sex in CSD in gut barrier development during their first days of life.

We also profiled sex-dependent gut microbial network features in first days' mice born by VD or CSD. Without accounting for sex, *Lactobacillus* was the key taxa centrality network of VD pups, while *Mucispirillum* for CSD pups.^47^ Here, NetMoss-based microbial network analysis,^58^ divided by sex, revealed an increased centrality network of butyrate producers in CSD animals, indicating a more interconnected microbial structure in their first days of life (Figure 4g–i). Among these, the butyrate-producing genera *Odoribacter, Alistipes, Lachnospiraceae (UCG-001* and *FCS020 group), [Eubacterium] siraeum group,* and *Ruminococcus* were enriched in CSD and exhibited high NetMoss scores, particularly *Alistipes*^59^ with the highest network centrality, independent of sex (Figure 4f,g).

Sex-specific butyrate-producers' signatures were detected in male CSD in the first days of life. They showed potential increased network connectivity for *Roseburia, Lachnospiraceae UCG-006, Anaerotruncus*, and *Butyricicoccus* compared to male VD counterparts (Figure 4e). *Roseburia* had the highest score. During childhood, it seems that a low relative abundance of *Roseburia* was associated with respiratory diseases^60^ and becomes more abundant only after weaning, with early food introduction promoting its enrichment in piglets. These results suggest that nutritional and developmental cues, such as those observed in CSD animals, can accelerate *Roseburia* establishment and abundance within the gut earlier than normal microbiota development.^61^

Also, a denser network centrality in butyrate-producing taxa (Figure 4g,i) was observed in male CSD. Unfortunately, butyrate levels were undetected during this period of life, limiting our conclusions regarding the functional production of butyrate.

### Males CSD had impaired colonic barrier function, low caecal butyrate levels, few remaining butyrate-producer taxa network centrality, and sex-specific abundant taxa during weaning

**Figure 5.**
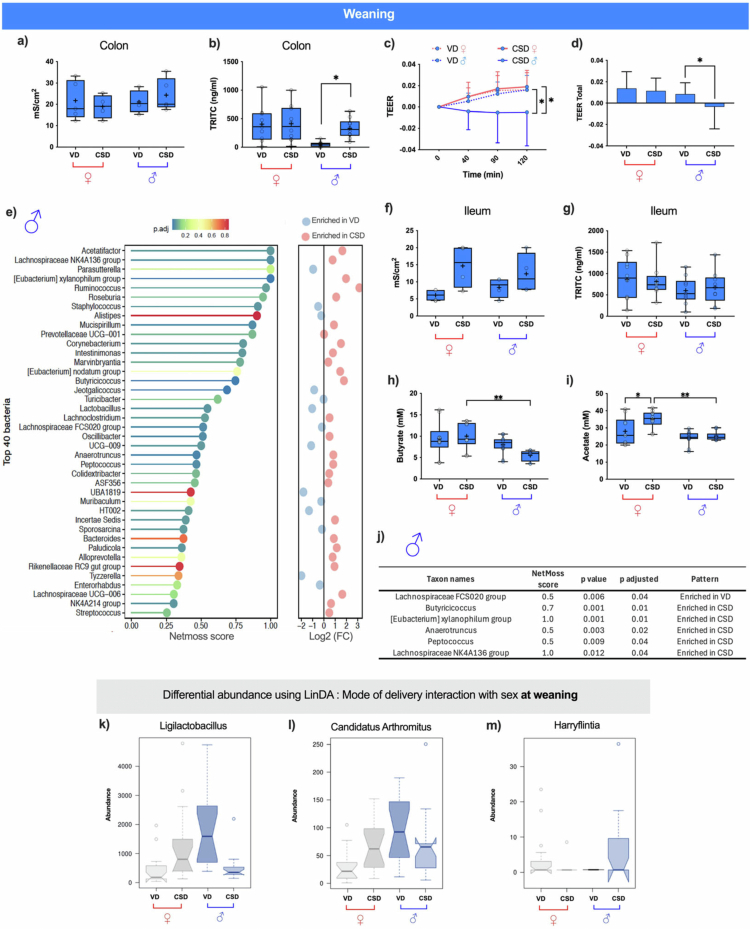
Sex effects interaction with delivery mode in gut taxa at weaning and delivery mode effects on epithelial barrier and microbial network at weaning depending on sex. Electrical conductance (mS/cm²) of colon (a) and ileal (f) tissues mounted in Ussing chambers, respectively, on females (♀, in red) and males (♂, in dark blue) born by vaginal (VD) or c-section (CSD) delivery during weaning (*N* = 5 animals per group). Tetramethylrhodamine isothiocyanate (TRITC) translocation in colon (b) and ileum (g) tissues mounted in Ussing chambers expressed in ng/mL. (c) Intestinal permeability measurements using transepithelial electrical resistance (TEER) and (d) total TEER variation over 120 min. (e) NetMoss scores of the top 40 microbial taxa by delivery mode at weaning in males. The fold change (FC) indicates the fold change in relative abundance. (h, i) Cecal acetate and butyrate levels in mM. (j) Butyrate-producers table with a NetMoss score higher or equal to 0.5 in males at weaning (*N* = 10 animals per group). (k–m) Boxplots with differential abundances of *Ligilactobacillus*, *Candidatus Arthromitus*, and *Harryflintia* interacting delivery mode with sex effects using LinDA analysis. Statistical comparisons were performed using Kruskal–Wallis tests for non-normally distributed data followed by Dunn's post hoc test and 2- or 3-way ANOVA for normal distribution, followed by Fisher's post hoc test. *p < 0.05, **p < 0.01.

Three gut barrier parameters impacted by CSD^47^ were assessed to evaluate sex effects altering CSD impact at weaning: (1) colonic and ileal epithelial conductance (Figure 5a,f), (2) colonic and ileal permeability measured by local tetramethylrhodamine isothiocyanate (TRITC) levels (Figure 5b,g), and (3) colonic transepithelial electrical resistance (TEER; Figure 5c,d). Caecal short-chain fatty acids (SCFAs) levels impacted in CSD animals during weaning^47^ were assessed by dividing the groups by sex (Figure 5h,i). Next, NetMoss score and linear models for differential abundance (LinDA) analyses were conducted to verify how sex interacts with the delivery mode shaping microbiota network centrality (Figure 5e,j) and differential abundance (Figure 5k–m), respectively.

A trend toward a delivery mode-by-tissue interaction in epithelial conductance was noted [*p* = 0.17; F(3, 30) = 11.4, *p* < 0.001]. No differences between groups after pairwise comparisons were found (Figure 5a-f). Paracellular permeability was strongly affected by sex (*p* < 0.001), with potential delivery-by-sex interactions (*p* = 0.17). An increase in paracellular permeability in CSD males compared to VD males was found after pairwise comparisons (Figure 5b). Finally, sex significantly influenced colonic TEER (*p* < 0.01; F(3, 75) = 3.1, *p* < 0.05), with a trend toward an interaction between delivery mode and sex (*p* = 0.08). When pairwise comparisons were made, a reduction in TEER was observed only in CSD males (Figure 5c). The total variation in resistance over 2 h was significantly reduced in CSD males [Figure 5d; F(3, 65) = 3.999, *p* < 0.01], which is consistent with impaired barrier maturation.

Caecal butyrate levels were significantly reduced in CSD males compared to VD males, while no reduction was observed in females (Figure 5h). In contrast, acetate concentrations were higher in CSD females compared to VD females, whereas male groups showed no increase (Figure 5i). These findings indicate that delivery mode alters SCFA profiles during weaning in a sex-dependent manner, with male CSD animals displaying reduced butyrate availability.

Gut microbial network connectivity was assessed using NetMoss score around weaning, and the strong central connectivity observed in CSD animals in their first days of life remained with fewer taxa only to CSD males (Figure 5e,k). Specifically, *Lachnospiraceae NK4A136* and *[Eubacterium] xylanophilum* taxa displayed greater centrality in CSD males, followed by the butyrate-producers *Butyricicoccus* and *Anaerotruncus*, which maintained centrality in CSD males' microbial network compared to their VD male counterparts (Figure 5e,k). Conversely, the non-butyrate-producer *Peptococcus* exhibited greater centrality in the CSD male networks. Curiously, the butyrate-producer *Lachnospiraceae FCS020* showed greater centrality in VD males' microbiota networks (Figure 5k), although in the first days of life, it had been more central in CSD animals (Figure 4g–i).

Finally, from LinDA integrative analysis, we observed some clear sex-dependent alterations around weaning in the abundance of the *Ligilactobacillus*, *Candidatus arthromitus*, and *Harryflintia* taxa (Figure 5h–j). Specifically, *Ligilactobacillus* and *Candidatus arthromitus* counts were higher in CSD females compared to VD females, whereas it was found decreased in CSD males compared to VD males, respectively (Figure 5h,i). Analysis at species levels also confirmed this pattern (Supl. Fig. 13). *Ligilactobacillus lactic acid* bacteria are usually related to benefits to the host,^62^ being decreased in CSD males. This is also the case for *Candidatus Arthromitus.*^63^ More present in the ileum, this bacterium is recognized by its protective immune function.^64^ The absence of CSD effects in females during weaning suggests a sex-driven counterbalance in the gut microbiota composition that may mitigate CSD-associated alterations in females, which are observed more clearly in males. *Harryflintia* was virtually absent in CSD females and VD males but significantly enriched in VD females and CSD males (Figure 5c). Owing to challenges in isolation, little is known about its effects on the host.^65^

### Key butyrate-producers from first days of life until young adulthood are sex- and delivery mode-dependent

**Figure 6.**
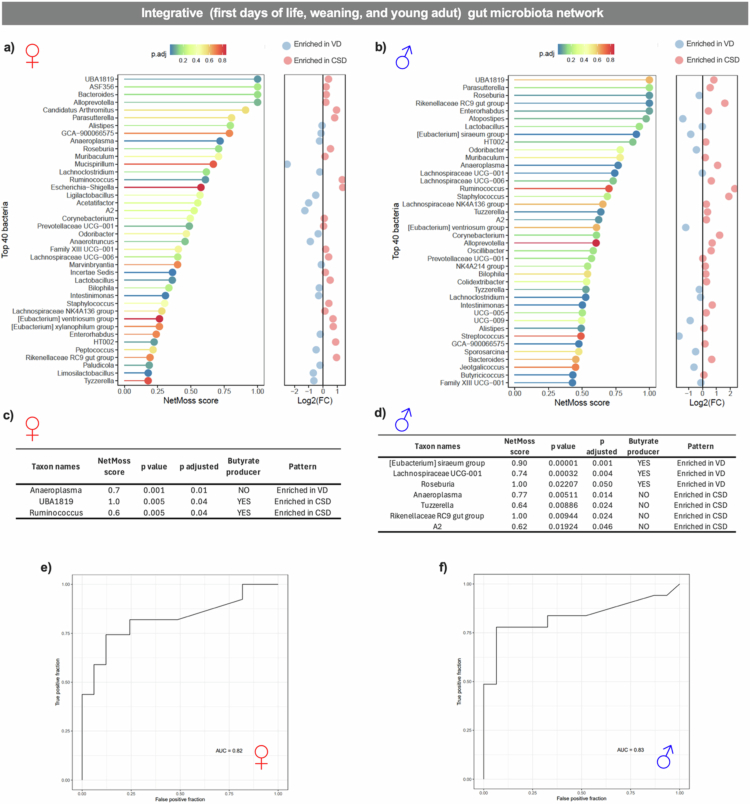
Key microbial taxa influencing the gut microbiota network in c-section vs. vaginal delivered females and males integrating first days of life until young age data. (a and b) Integrated NetMoss score for evaluating the microbial network in females and males, respectively. (c and d) Table with key butyrate producers or non-butyrate-producer genes influencing the gut microbiota network in c-section (CSD) and vaginal delivery (VD) animals. (e and f) ROC curve analysis revealing that key microbial taxa had an area under the curve (AUC) higher than 0.8, effectively distinguishing CSD from VD animals. *N* = 20 animals per group.

To explore microbial network centrality that might be related to the increased susceptibility to colitis reoccurrence observed in CSD males during adulthood, we examined whether the establishment and persistence of butyrate-producing taxa trajectories across development differed according to delivery mode and sex. During the first days of life, CSD animals exhibited butyrate producers' network centrality,^47^ with males having more butyrate producers on their network centrality (Figure 4f–i), whereas at weaning, this pattern was restricted to CSD males (Figure 5e,k). Around the same period, sex-dependent effects interacting with CSD were also detected in the differential abundance of some taxa (Figure 5g–i). These observations suggest that delivery mode, sex, and age jointly influence the establishment of butyrate producers and their connectivity during early development. To evaluate the key taxa network across early development, *i.e.,* between the first days of life until young adulthood, we used NetMoss to identify sex-dependent key taxa in males and females in VD and CSD animals (Figure 6).

In females, some taxa showed high NetMoss scores enriched between VD and CSD animals across ages (Figure 6a). Among them, one non-butyrate-producer (*Anaeroplasma*, score = 0.7) was distinguished as a key taxon enriched in VD. In contrast, the butyrate-producers *UBA1819* (score = 1.0) and *Ruminococcus* (score = 0.6) were the key taxa enriched in CSD female animals (Figure 6c).

In males, distinct taxa also separated VD from CSD animals (Figure 6b). Opposite to the pattern in females, butyrate-producers were the key enriched taxa in VD, including *[Eubacterium] siraeum group* (score = 0.9), *Lachnospiraceae UCG-001* (score = 0.7), and *Roseburia* (score = 1.0). Conversely, the non-butyrate-producers *Anaeroplasma* (score = 0.77), *Tuzzerella* (score = 0.64), *Rikenellaceae RC9 gut group* (score = 1.0), and *A2* (score = 0.62) were the key taxa enriched in male CSD animals (Figure 6d). Microbial key signatures in both females (AUC = 0.82) and males (AUC = 0.83) were robustly discriminated in both CSD and VD groups (Figure 6e–f).

These results highlight that CSD leads to age- and sex-dependent shifts in butyrate-producing taxa abundance and their cooccurring patterns that may impact immunity imprinting, gut barrier feature establishment, and colitis severity. The key taxa in CSD females across early development were butyrate producers (Figure 6a,c) with the opposite profile in VD animals but not in CSD males (Figure 6b,d), clearly exhibiting the complexity of the gut microbial network across development in CSD animals and the impact of sex.

### Differential microbial abundance analysis across life stages until adulthood reveals a transition from stronger early delivery-mode to later sex associated effects in gut microbiota

To dissect how the relative influence of delivery mode and sex evolved across development and potential cues linking sex-dependent colitis reoccurrence susceptibility, we performed differential microbial abundance analyses using LinDA, which is specifically designed for compositional microbiome data and correct the bias typically introduced by traditional linear regression models that are not based on centered log-ratio (CLR)–transformed abundances.

**Figure 7.**
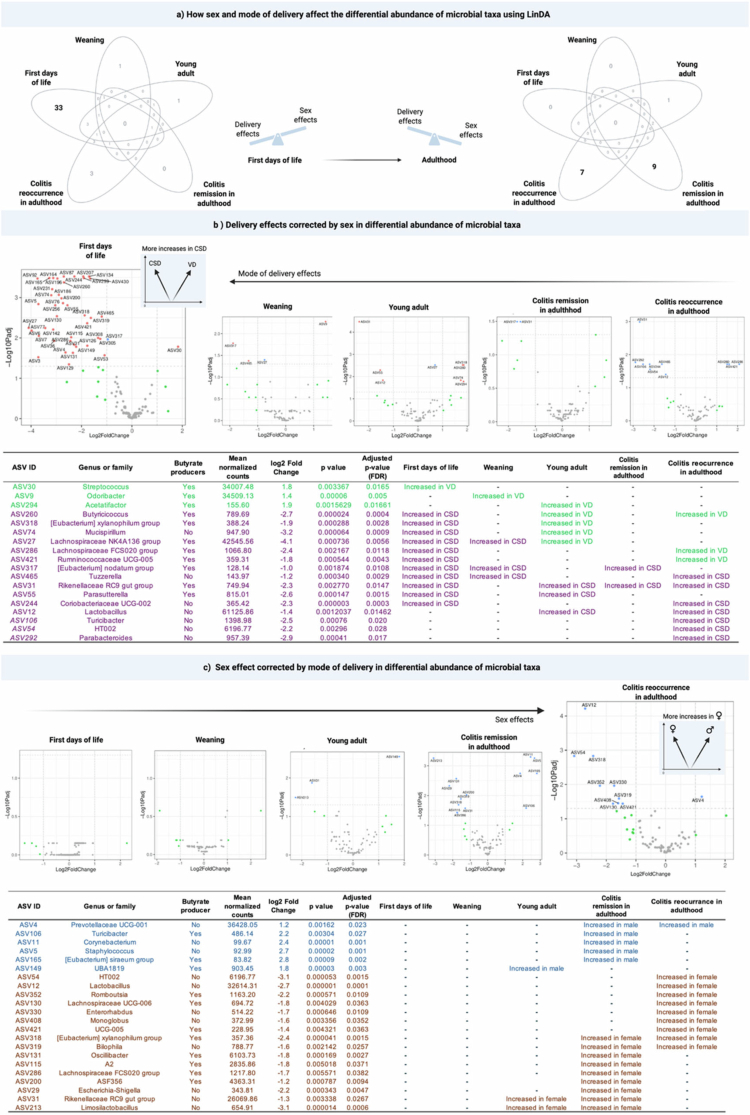
Effects of delivery mode and sex on the differential abundance of microbial taxa during life stages. (a) Venn diagrams summarizing the number of differentially abundant taxa associated with delivery mode corrected by sex (left) or sex corrected by delivery mode (right) across five life stages: first days of life, weaning, young *adult*, *colitis remission in adulthood*, and colitis reoccurrence in adulthood. Early differences were driven primarily by delivery mode, whereas sex effects emerged with age. (b) Volcano plots showing microbial taxa differentially abundant between CSD and VD animals after correcting for sex at each life stage. Insets highlight the overall direction of change (more increases in CSD or VD). Representative taxa are labeled and significantly altered ASVs are listed in the accompanying table, including their genus/family assignment, butyrate-producing capacity, mean normalized counts, log2-fold-change, *p*-values, and life stage where differences were detected. (c) Volcano plots showing microbial taxa differentially abundant between males and females after correction for delivery mode at each life stage. Insets indicate the overall direction of change (more increases in males or females). Significantly altered ASVs are detailed in the accompanying table with taxonomic assignment, butyrate-producing capacity, statistical values, and stage-specific differences. See Supl. Figures 3–11 for further analysis details.

Overall, LinDA-based analyses revealed a clear developmental transition in the determinants of microbial abundance composition from stronger early effects driven by delivery mode to later patterns increasingly shaped by sex. Venn diagrams highlight this taxa transition between delivery-associated (corrected for sex) and sex-associated (corrected for delivery) taxa across the first days of life, weaning, postweaning, young adulthood, colitis remission, and colitis reoccurrence in adulthood (Figure 7a).

In the first days of life, most differentially abundant taxa were associated with delivery mode, with no sex effect associations observed (Figure 7b,c). The results revealed that *Streptococcus*, *Odoribacter*, and *Acetatifactor* were more abundant in VD animals at distinct stages (first days, weaning, and young adulthood, respectively). These genera are nonspore-forming butyrate producers, with *Streptococcus* and *Odoribacter* being more abundant than *Acetatifactor* in normalized counts. In contrast, the majority of taxa more abundant in CSD animals during early life belonged to Firmicutes and Bacteroidota, particularly Oscillospiraceae (12 taxa), Lachnospirales (13), and Bacteroidales (6), including Oscillospiraceae (6), Ruminococcaceae (6), and Lachnospiraceae (13), at the family level. Most of these differentially abundant taxa were butyrate producers (see Supl. Table 3 for further details), indicating that among the taxa enriched early in life, CSD animals harbored more and broader varieties of abundant butyrate producers than VD animals.

As the animals aged, this broader representation of butyrate producers with increased abundancy in CSD animals diminished. Some taxa initially enriched in CSD later became more abundant in VD animals, including *Butyricicoccus*, *[Eubacterium] xylanophilum group*, *Mucispirillum*, *Lachnospiraceae NK4A136*, *Lachnospiraceae FCS020*, and *Ruminococcaceae UCG-005*. Except for *Mucispirillum*, all are butyrate producers.

By adulthood, CSD animals displayed a greater differential abundance in a variety of non–butyrate-producing taxa. Interestingly, taxa enriched in VD animals were life stage-specific and transient, whereas some taxa enriched in CSD animals persisted across stages. In particular, the non-butyrate-producer *Rikenellaceae RC9 gut* group remained consistently enriched in CSD animals at multiple stages, except weaning. Giving support, LinDA analysis at species levels maintains *Rikenellaceae RC9 gut* group as the most enriched at multiple stages, except during weaning and the colitis remission period in adulthood (Supl. Table 4).

When analyses were corrected for delivery mode, no sex-associated taxa were identified until young adulthood (Figure 7c). This turning point coincides with the onset of sexual hormone elevation in mice.^66^ The number of sex-associated taxa with higher abundance increased further into adulthood, with females showing a greater representation than males.

Colitis reoccurrence reduced the number of CSD-corrected abundant taxa in males compared to colitis remission, leaving *Prevotellaceae UCG-001* as the only persistent enrichment at the genus (Figure 7c) and species levels (Supl. Table 4). Almost all enriched taxa in females shifted from colitis remission to reoccurrence, with the enrichment persistence of *[Eubacterium] xylanophilum group* and *Biophila* only. Curiously, in agreement with the network inferences, this shift was marked by more abundant butyrate producers in colitis remission taxa to less in colitis reoccurrence in females compared to males in the same period (Figure 7c). Worth mentioning, females had more abundant *Lactobacillus johnsonii* during colitis reoccurrence in adulthood, specifically. *L. johnsonii* is usually considered beneficial to the host, such as decreasing colitis,^67^ which might be related to the lower colitis reoccurrence in females (Figure 6).

## Discussion

This study reveals that delivery mode and sex seem to interact to shape microbiota–gut barrier crosstalk across life, with unique life stage-specific trajectories that reflect the dynamic co-development of the host and microbiota. By integrating classical physiological, immunological, and microbial network analyses, we show that cesarean delivery (CSD) might induce sex-dependent alterations in gut barrier function and microbiota composition that persist from early life into adulthood. These effects appear more pronounced in males, who display increased susceptibility to colitis reoccurrence later in life, along with early-life microbial and barrier alterations. Together, these observations highlight the potential necessity of considering both delivery mode and sex when investigating early-life programming of host–microbiota interactions and their potential long-term consequences for inflammatory disease susceptibility.

These observations may be relevant in light of human studies reporting an association between CSD and an increased risk of inflammatory bowel diseases.^22^^-^^24^ Although evidence remains heterogeneous, some studies have suggested sex-related differences.^45^^,^^46^ While these findings cannot fully establish causality, they support the hypothesis that delivery mode influences early-life microbiota trajectories and host responses in a sex-dependent manner, potentially contributing to differences in disease susceptibility later in life. Early-life microbial network butyrate-producing taxa were prominent in CSD animals during the first days of life but declined by weaning, suggesting dynamic reorganization of microbial interactions across development. When microbial profiles from multiple developmental stages were considered together, integrated LinDA differential abundance and NetMoss network centrality analyses revealed that the microbial community structure evolved from delivery mode–driven differentiation in early life toward increasingly sex-dependent patterns during maturation. This transition likely reflects the influence of sex-specific host regulation,^68^^,^^69^ including immune, metabolic, and hormonal factors that emerge during development.

Further research is needed to better understand the developmental shift observed in microbial network centrality. In particular, it remains unclear how the key non–butyrate-producing taxa identified in male CSD networks and the butyrate-producing taxa enriched in female CSD networks interact with the microbial communities detected at earlier developmental stages. The differences observed between integrated network analyses and those performed at individual time points suggest that microbial interactions evolve dynamically across development.

Interestingly, when microbial data from multiple developmental stages were integrated, distinct microbial signatures robustly distinguished CSD from VD animals. This observation highlights the potential value of integrative network approaches for identifying persistent microbial features associated with delivery mode and underscores the importance of examining microbial interactions across developmental time rather than focusing on isolated life stages.

A particularly notable taxon emerging across analyses was the *Rikenellaceae RC9 gut group*, a non–butyrate-producer that was consistently enriched in CSD animals across most developmental stages, suggesting that this taxon may represent a persistent microbial feature associated with delivery mode. In parallel, network analyses revealed that RC9 occupied a highly central position within microbial interaction networks, particularly in males, indicating that this taxon may play an important structural role within the CSD-associated microbial community. Previous studies portray *RC9* as context-dependent with beneficial effects in some settings,^70^^,^^71^ yet detrimental in others.^72^^-^^75^ Notably, *Rikenellaceae RC9 gut group* has been reported to show negative correlation with butyrate levels and positive correlations with butyrate derivatives, suggesting potential competition with butyrate producers for shared substrates.^76^ Additional evidence implicates *RC9* in lipid metabolism.^77^^,^^78^ Higher *Rikenellaceae RC9 gut group* abundance has been associated with increased body weight and fat mass^77^^,^^78^ or increased subcutaneous adipose tissue.^79^

This study also acknowledges some limitations. First, the modest sample sizes and intragroup variability at certain timepoints and attritions (Sup. Tables 1 and 2) may have reduced our power to detect subtle sex differences. Second, the lack of estrous cycle monitoring. Fluctuations in ovarian hormones across the cycle can influence female physiological outcomes, and the absence of cycle monitoring by vaginal smears could have introduced additional variability, thus masking sex-specific effects in females. Third, circulating sex hormone levels were not measured, preventing us from directly correlating the observed sex differences to hormonal status or from accurately defining the stage of puberty. Fourth, microbiota analyses were based on taxa abundance and microbial network inference, which does not necessarily reflect their metabolic production, such as SCFA synthesis, and functional validation is needed to confirm these associations. Therefore, the functional implications of the identified taxa and network alterations remain partially inferential. Additional functional validation would be needed to confirm these associations for each stage of life, and experiments such as fecal microbiota transplantation (FMT) or cohousing studies would be necessary to determine whether these microbial features causally contribute to the observed barrier dysfunction and increased disease susceptibility later in life. Finally, all the experiments were performed in a single inbred mouse strain, limiting the generalizability of all the results.

Nevertheless, the consistent patterns across independent analyses and life stages underscore our conclusions. Using new approaches to stratify data and assess sex effects, we reinforce the possibility that delivery mode programs sex-dependent trajectories of host–microbe interactions, with long-lasting consequences for gut barrier fitness and colitis susceptibility more prominent in males. The integration of classical and compositional differential abundance analyses, LinDA linear modeling and NetMoss network profiling uncovered subtle interactions between sex and delivery mode that would be missed by abundance-based methods alone. These results are in line with the potential need to design early-life microbiota-targeted strategies—potentially personalized by sex—to mitigate the long-term health effects of C-section delivery.

## Supplementary Material

Supl._Figures_Legend.docx Supplemental Material

Supl_tables_REVISED.docx Supplemental Material

Sup. Figures Sup. Fig. 1

## Data Availability

This work re-analyzed pre-clinical parameters originally reported as differing between cesarean-delivered (CSD) and vaginally delivered (VD) animals, now stratified by sex.^47^ Raw microbial 16S rRNA gene sequencing data available in the NCBI Sequence Read Archive under BioProject PRJNA876103 (accessions SAMN30638467–SAMN30638852). Other data are available from the corresponding author upon reasonable request.

## References

[1] Koceva A, Herman R, Janez A, Rakusa M, Jensterle M. Sex- and gender-related differences in obesity: from pathophysiological mechanisms to clinical implications. Int J Mol Sci. 2024;25(13):7342. doi: 10.3390/ijms25137342. 39000449 PMC11242171

[2] Liu DS, Wang X, Zhong X, Cao H, Zhang F. Sexual dimorphism in the gut microbiota and sexual dimorphism in chronic diseases: association or causation? J Steroid Biochem Mol Biol. 2024;237:106451. doi: 10.1016/j.jsbmb.2023.106451. 38154505

[3] Clocchiatti A, Cora E, Zhang Y, Dotto GP. Sexual dimorphism in cancer. Nat Rev Cancer. 2016;16(5):330–339. doi: 10.1038/nrc.2016.30. 27079803

[4] Goodman WA, Erkkila IP, Pizarro TT. Sex matters: impact on pathogenesis, presentation and treatment of inflammatory bowel disease. Nat Rev Gastroenterol Hepatol. 2020;17(12):740–754. doi: 10.1038/s41575-020-0354-0. 32901108 PMC7750031

[5] Ober C, Loisel DA, Gilad Y. Sex-specific genetic architecture of human disease. Nat Rev Genet. 2008;9(12):911–922. doi: 10.1038/nrg2415. 19002143 PMC2694620

[6] Gao A, Su J, Liu R, Zhao S, Li W, Xu X, Shi J, Gu B, Zhang J, Wang X, et al. Sexual dimorphism in glucose metabolism is shaped by androgen-driven gut microbiome. Nat Commun. 2021;12(1):7080. doi: 10.1038/s41467-021-27187-7. 34873153 PMC8648805

[7] Borgo F, Garbossa S, Riva A, Severgnini M, Luigiano C, Benetti A, Pontiroli AE, Morace G, Borghi E. Body mass index and sex affect diverse microbial niches within the gut. Front Microbiol. 2018;9:213. doi: 10.3389/fmicb.2018.00213. 29491857 PMC5817072

[8] Falony G, Joossens M, Vieira-Silva S, Wang J, Darzi Y, Faust K, Kurilshikov A, Bonder MJ, Valles-Colomer M, Vandeputte D, et al. Population-level analysis of gut microbiome variation. Science. 2016;352(6285):560–564. doi: 10.1126/science.aad3503. 27126039

[9] Sisk-Hackworth L, Kelley ST, Thackray VG. Sex, puberty, and the gut microbiome. Reproduction. 2023;165(2):R61–R74. doi: 10.1530/REP-22-0303. 36445259 PMC9847487

[10] Ma X, Ding J, Ren H, Xin Q, Li Z, Han L, Liu D, Zhuo Z. Distinguishable influence of the delivery mode, feeding pattern, and infant sex on dynamic alterations in the intestinal microbiota in the first year of life. MicEc. 2023;86(3):1799–1813. doi: 10.1007/s00248-023-02188-9. 36864279

[11] Neu J, Rushing J. Cesarean versus vaginal delivery: long-term infant outcomes and the hygiene hypothesis. Clin Perinatol. 2011;38(2):321–331. doi: 10.1016/j.clp.2011.03.008. 21645799 PMC3110651

[12] Rebelo F, Da Rocha CMM, Cortes TR, Dutra CL, Kac G. High cesarean prevalence in a national population-based study in Brazil: the role of private practice. Acta Obstet Gynecol Scand. 2010;89(7):903–908. doi: 10.3109/00016349.2010.484044. 20583936

[13] Althabe F, Sosa C, Belizán JM, Gibbons L, Jacquerioz F, Bergel E. Cesarean section rates and maternal and neonatal mortality in low-, medium-, and high-income countries: an ecological study. Birth. 2006;33(4):270–277. doi: 10.1111/j.1523-536X.2006.00118.x. 17150064

[14] Betran AP, Torloni MR, Zhang J, Ye J, Mikolajczyk R, Deneux-Tharaux C, Oladapo OT, Souza JP, Tunçalp Ö, Vogel JP, et al. What is the optimal rate of caesarean section at population level? A systematic review of ecologic studies. Reprod Health. 2015;12:57. doi: 10.1186/s12978-015-0043-6. 26093498 PMC4496821

[15] Betran AP, Ye J, Moller A, Souza JP, Zhang J. Trends and projections of caesarean section rates: global and regional estimates. BMJ Glob Health. 2021;6(6):e005671. doi: 10.1136/bmjgh-2021-005671. PMC8208001 34130991

[16] Eggesbo M, Eggesbø M, Botten G, Stigum H, Nafstad P, Magnus P. Is delivery by cesarean section a risk factor for food allergy? J Allergy Clin Immunol. 2003;112(2):420–426. doi: 10.1067/mai.2003.1610. 12897751

[17] Laubereau B, Filipiak-Pittroff B, von Berg A, Grübl A, Reinhardt D, Wichmann E, Koletzko S, GINI Study Group. Caesarean section and gastrointestinal symptoms, atopic dermatitis, and sensitisation during the first year of life. Arch Dis Child. 2004;89(11):993–997. doi: 10.1136/adc.2003.043265. 15499049 PMC1719727

[18] Bager P, Wohlfahrt J, Westergaard T. Caesarean delivery and risk of atopy and allergic disesase: meta-analyses. Clin Exp Allergy. 2008;38(4):634–642. doi: 10.1111/j.1365-2222.2008.02939.x. 18266879

[19] Huh SY, Rifas-Shiman SL, Zera CA, Edwards JWR, Oken E, Weiss ST, Gillman MW. Delivery by caesarean section and risk of obesity in preschool age children: a prospective cohort study. Arch Dis Child. 2012;97(7):610–616. doi: 10.1136/archdischild-2011-301141. 22623615 PMC3784307

[20] Pei Z, Heinrich J, Fuertes E, Flexeder C, Hoffmann B, Lehmann I, Schaaf B, von Berg A, Koletzko S. Cesarean delivery and risk of childhood obesity. J Pediatr. 2014;164(5):1068–1073 e2. doi: 10.1016/j.jpeds.2013.12.044. 24508442

[21] Blustein J, Attina T, Liu M, Ryan AM, Cox LM, Blaser MJ, Trasande L. Association of caesarean delivery with child adiposity from age 6 weeks to 15 years. Int J Obes (Lond). 2013;37(7):900–906. doi: 10.1038/ijo.2013.49. 23670220 PMC5007946

[22] Li Y, Tian Y, Zhu W, Gong J, Gu L, Zhang W, Guo Z. Cesarean delivery and risk of inflammatory bowel disease: a systematic review and meta-analysis. Scand J Gastroenterol. 2014;49(7):834–844. doi: 10.3109/00365521.2014.910834. 24940636

[23] Bruce A, Black M, Bhattacharya S. Mode of delivery and risk of inflammatory bowel disease in the offspring: systematic review and meta-analysis of observational studies. Inflamm Bowel Dis. 2014;20(7):1217–1226. doi: 10.1097/MIB.0000000000000075. 24874459

[24] Bager P, Simonsen J, Nielsen NM, Frisch M. Cesarean section and offspring's risk of inflammatory bowel disease: a national cohort study. Inflamm Bowel Dis. 2012;18(5):857–862. doi: 10.1002/ibd.21805. 21739532

[25] Mueller NT, Whyatt R, Hoepner L, Oberfield S, Dominguez-Bello MG, Widen EM, Hassoun A, Perera F, Rundle A. Prenatal exposure to antibiotics, cesarean section and risk of childhood obesity. Int J Obes (Lond). 2015;39(4):665–670. doi: 10.1038/ijo.2014.180. 25298276 PMC4390478

[26] Maaser C, Langholz E, Gordon H, Burisch J, Ellul P, Hernández Ramirez V, Karakan T, Katsanos KH, Krustins E, Levine A, et al. European Crohn's and colitis organisation topical review on environmental factors in IBD. J Crohns Colitis. 2016:jjw223. doi: 10.1093/ecco-jcc/jjw223. 28039310

[27] Hviid A, Svanstrom H, Frisch M. Antibiotic use and inflammatory bowel diseases in childhood. Gut. 2011;60(1):49–54. doi: 10.1136/gut.2010.219683. 20966024

[28] Zhong Z, Chen M, Dai S, Wang Y, Yao J, Shentu H, Huang J, Yu C, Zhang H, Ren W. Association of cesarean section with asthma in children/adolescents: a systematic review and meta-analysis based on cohort studies. BMC Pediatr. 2023;23(1):571. doi: 10.1186/s12887-023-04396-1. 37974127 PMC10652517

[29] Liang Y, Zhang J, Bai S, Du S, Yang X, Wang Z. Short-term and long-term effects of cesarean section on asthma and wheezing: a cohort study and meta-analysis. Respir Med. 2023;215:107300. doi: 10.1016/j.rmed.2023.107300. 37257787

[30] Maynard CL, Elson CO, Hatton RD, Weaver CT. Reciprocal interactions of the intestinal microbiota and immune system. Nature. 2012;489(7415):231–241. doi: 10.1038/nature11551. 22972296 PMC4492337

[31] Bokulich NA, Chung J, Battaglia T, Henderson N, Jay M, Li H, Lieber A, Wu F, Perez-Perez GI, Chen Y, et al. Antibiotics, birth mode, and diet shape microbiome maturation during early life. Sci Transl Med. 2016;8(343):343–382. doi: 10.1126/scitranslmed.aad7121. PMC5308924 27306664

[32] Yoon DY, Mansukhani NA, Stubbs VC, Helenowski IB, Woodruff TK, Kibbe MR. Sex bias exists in basic science and translational surgical research. Surgery. 2014;156(3):508–516. doi: 10.1016/j.surg.2014.07.001. 25175501

[33] Melloni C, Berger JS, Wang TY, Gunes F, Stebbins A, Pieper KS, Dolor RJ, Douglas PS, Mark DB, Newby LK. Representation of women in randomized clinical trials of cardiovascular disease prevention. Circ Cardiovasc Qual Outcomes. 2010;3(2):135–142. doi: 10.1161/CIRCOUTCOMES.110.868307. 20160159

[34] Mazure CM, Jones DP. Twenty years and still counting: including women as participants and studying sex and gender in biomedical research. BMC Womens Health. 2015;15:94. doi: 10.1186/s12905-015-0251-9. 26503700 PMC4624369

[35] Solopov P, Colunga Biancatelli RML, Dimitropoulou C, Catravas JD. Sex-related differences in murine models of chemically induced pulmonary fibrosis. Int J Mol Sci. 2021;22(11):5909. doi: 10.3390/ijms22115909. 34072833 PMC8198091

[36] Orschell CM, Wu T, Patterson AM. Impact of age, sex, and genetic diversity in murine models of the hematopoietic acute radiation syndrome (H-ARS) and the delayed effects of acute radiation exposure (DEARE). Curr Stem Cell Rep. 2022;8(3):139–149. doi: 10.1007/s40778-022-00214-z. 36798890 PMC9928166

[37] Vichaya EG, Ford BG, Moltenkine JM, Taniguchi CM, Phillip West A, Dantzer R. Sex differences in the behavioral and immune responses of mice to tumor growth and cancer therapy. Brain Behav Immun. 2021;98:161–172. doi: 10.1016/j.bbi.2021.08.225. 34418499 PMC8511067

[38] Bernardi JR, Pinheiro TV, Mueller NT, Sueno Goldani HA, Pereira Gutierrez MR, Bettiol H, Moura da Silva AA, Antônio Barbieri M, Zubaran Goldani M. Cesarean delivery and metabolic risk factors in young adults: a Brazilian birth cohort study. AJCN. 2015;102(2):295–301. doi: 10.3945/ajcn.114.105205. PMC6546227 26085513

[39] Hansen S, Halldorsson TI, Olsen SF, Rytter D, Bech BH, Granström C, Henriksen TB, Chavarro JE. Birth by cesarean section in relation to adult offspring overweight and biomarkers of cardiometabolic risk. Int J Obes (Lond). 2018;42(1):15–19. doi: 10.1038/ijo.2017.175. 28757643

[40] Mínguez-Alarcón L, Rifas-Shiman SL, Mitchell C, Sordillo J, Aris IM, Hivert M, Oken E, Chavarro JE. Cesarean delivery and metabolic health and inflammation biomarkers during mid-childhood and early adolescence. Pediatr Res. 2022;91(3):672–680. doi: 10.1038/s41390-021-01503-9. 33824455 PMC8492770

[41] Bridgman SL, Penfold S, Field CJ, Haqq AM, Mandhane PJ, Moraes TJ, Turvey SE, Simons E, Subbarao P, Kozyrskyj AL. Pre-labor and post-labor cesarean delivery and early childhood adiposity in the Canadian healthy infant longitudinal development (CHILD) cohort study. Int J Obes (Lond). 2024;48(5):717–724. doi: 10.1038/s41366-024-01480-z. 38302592

[42] Mueller NT, Zhang M, Rifas-Shiman SL, Oken E, Hivert M, Chavarro J. Mode of delivery, type of labor, and measures of adiposity from childhood to teenage: project viva. Int J Obes (Lond). 2021;45(1):36–44. doi: 10.1038/s41366-020-00709-x. 33199815 PMC7755743

[43] Zhang S, Zhou J, Yang M, Tao X, Huang K. Sex-specific association between elective cesarean section and growth trajectories in preschool children: a prospective birth cohort study. Front Public Health. 2022;10:985851. doi: 10.3389/fpubh.2022.985851. 36203696 PMC9530938

[44] Yoshida T, Matsumura K, Hatakeyama T, Inadera H, Kamijima M, Yamazaki S, Ohya Y, Kishi R, Yaegashi N, Hashimoto K, et al. Association between cesarean section and neurodevelopmental disorders in a Japanese birth cohort: the Japan environment and Children's study. BMC Pediatr. 2023;23(1):306. doi: 10.1186/s12887-023-04128-5. 37331958 PMC10278360

[45] Malmborg P, Bahmanyar S, Grahnquist L, Hildebrand H, Montgomery S. Cesarean section and the risk of pediatric Crohn's disease. Inflamm Bowel Dis. 2012;18(4):703–708. doi: 10.1002/ibd.21741. 21538718

[46] Andersen V, Erichsen R, Frøslev T, Sørensen HT, Ehrenstein V. Differential risk of ulcerative colitis and Crohn's disease among boys and girls after cesarean delivery. Inflamm Bowel Dis. 2013;19(1):E8–E10. doi: 10.1002/ibd.22841. 22147542

[47] Barone M, Ramayo-Caldas Y, Estellé J, Tambosco K, Chadi S, Maillard F, Gallopin M, Planchais J, Chain F, Kropp C, et al. Gut barrier-microbiota imbalances in early life lead to higher sensitivity to inflammation in a murine model of C-section delivery. Microbiome. 2023;11(1):140. doi: 10.1186/s40168-023-01584-0. 37394428 PMC10316582

[48] Barone M, Chain F, Sokol H, Brigidi P, Bermúdez-Humarán LG, Langella P, Martín R. A versatile new model of chemically induced chronic colitis using an outbred murine strain. Front Microbiol. 2018;9:565. doi: 10.3389/fmicb.2018.00565. 29636738 PMC5881104

[49] Martin R, Martín R, Chamignon C, Mhedbi-Hajri N, Chain F, Derrien M, Escribano-Vázquez U, Garault P, Cotillard A, Pham HP, et al. The potential probiotic *Lactobacillus rhamnosus* CNCM I-3690 strain protects the intestinal barrier by stimulating both mucus production and cytoprotective response. Sci Rep. 2019;9(1):5398. doi: 10.1038/s41598-019-41738-5. 30931953 PMC6443702

[50] Perrier C CB. Gut permeability and food allergies. Clin Exp Allergy. 2011;41(1):20–28. doi: 10.1111/j.1365-2222.2010.03639.x. 21070397

[51] Wrzosek L, Miquel S, Noordine M, Bouet S, Chevalier-Curt MJ, Robert V, Philippe C, Bridonneau C, Cherbuy C, Robbe-Masselot C, et al. Bacteroides thetaiotaomicron and *Faecalibacterium prausnitzii* influence the production of mucus glycans and the development of goblet cells in the colonic epithelium of a gnotobiotic model rodent. BMC Biol. 2013;11:61. doi: 10.1186/1741-7007-11-61. 23692866 PMC3673873

[52] Callahan BJ, McMurdie PJ, Rosen MJ, Han AW, Johnson AJA, Holmes SP. DADA2: high-resolution sample inference from illumina amplicon data. Nat Methods. 2016;13(7):581–583. doi: 10.1038/nmeth.3869. 27214047 PMC4927377

[53] McMurdie PJ, Holmes S. Phyloseq: an R package for reproducible interactive analysis and graphics of microbiome census data. PLoS One. 2013;8(4):e61217. doi: 10.1371/journal.pone.0061217. 23630581 PMC3632530

[54] Li H, Sheng D, Jin C, Zhao G, Zhang L. Identifying and ranking causal microbial biomarkers for colorectal cancer at different cancer subsites and stages: a mendelian randomization study. Front Oncol. 2023;13:1224705. doi: 10.3389/fonc.2023.1224705. 37538123 PMC10395834

[55] Scardoni G, Tosadori G, Faizan M, Spoto F, Fabbri F, Laudanna C. Biological network analysis with CentiScaPe: centralities and experimental dataset integration. F1000Res. 2014;3:139. doi: 10.12688/f1000research.4477.1. 26594322 PMC4647866

[56] Zhou H, He K, Chen J, Zhang X. LinDA: linear models for differential abundance analysis of microbiome compositional data. Genome Biol. 2022;23(1):95. doi: 10.1186/s13059-022-02655-5. 35421994 PMC9012043

[57] Xia Y, Liu C, Li R, Zheng M, Feng B, Gao J, Long X, Zuo X. Lactobacillus-derived indole-3-lactic acid ameliorates colitis in cesarean-born offspring via activation of aryl hydrocarbon receptor. iSci. 2023;26(11):108279. doi: 10.1016/j.isci.2023.108279. PMC10656274 38026194

[58] Xiao L, Zhang F, Zhao F. Large-scale microbiome data integration enables robust biomarker identification. Nat Comput Sci. 2022;2(5):307–316. doi: 10.1038/s43588-022-00247-8. 38177817 PMC10766547

[59] Parker BJ, Wearsch PA, Veloo ACM, Rodriguez-Palacios A. The Genus Alistipes: Gut Bacteria With Emerging Implications to Inflammation, Cancer, and Mental Health. Front Immunol. 2020;11:906. doi: 10.3389/fimmu.2020.00906. 32582143 PMC7296073

[60] Alcazar CG, Paes VM, Shao Y, Oesser C, Miltz A, Lawley TD, Brocklehurst P, Rodger A, Field N. The association between early-life gut microbiota and childhood respiratory diseases: a systematic review. Lancet Microbe. 2022;3(11):e867–e880. doi: 10.1016/S2666-5247(22)00184-7. 35988549 PMC10499762

[61] Choudhury R, Middelkoop A, Boekhorst J, Gerrits WJJ, Kemp B, Bolhuis JE, Kleerebezem M. Early life feeding accelerates gut microbiome maturation and suppresses acute post-weaning stress in piglets. Environ Microbiol. 2021;23(11):7201–7213. doi: 10.1111/1462-2920.15791. 34655283 PMC9291500

[62] Yang Y, Song X, Wang G, Xia Y, Xiong Z, Ai L. Understanding Ligilactobacillus salivarius from Probiotic Properties to Omics Technology: A Review. Foods. 2024;13(6):895. doi: 10.3390/foods13060895. 38540885 PMC10969406

[63] Bolotin A, de Wouters T, Schnupf P, Bouchier C, Loux V, Rhimi M, Jamet A, Dervyn R, Boudebbouze S, Blottière HM, et al. Genome sequence of "Candidatus Arthromitus" sp. Strain SFB-Mouse-NL, a commensal bacterium with a key role in postnatal maturation of gut immune functions. Genome Announc. 2014;2(4), 10.1128/genomeA.00705-14. PMC4102870 25035333

[64] Lécuyer E, Rakotobe S, Lengliné-Garnier H, Lebreton C, Picard M, Juste C, Fritzen R, Eberl G, McCoy KD, Macpherson AJ, et al. Segmented filamentous bacterium uses secondary and tertiary lymphoid tissues to induce gut IgA and specific T helper 17 cell responses. Immunity. 2014;40(4):608–620. doi: 10.1016/j.immuni.2014.03.009. 24745335

[65] Petzoldt H. Harryflintia acetispora gen. nov., sp. nov., isolated from chicken caecum. Int J Syst Evol Microbiol . 2016.;66:4099–4104. doi: 10.1099/ijsem.0.001317. 27432404

[66] Bell MR. Comparing postnatal development of gonadal hormones and associated social behaviors in rats, mice, and humans. Endocrinology. 2018;159(7):2596–2613. doi: 10.1210/en.2018-00220. 29767714 PMC6692888

[67] Liu HY, Yuan P, Li S, Ogamune KJ, Shi X, Zhu C, Ennab W, Hu P, Ahmed AA, Zhang Y, et al. Lactobacillus johnsonii alleviates experimental colitis by restoring intestinal barrier function and reducing NET-mediated gut-liver inflammation. Commun Biol. 2025;8(1):1222. doi: 10.1038/s42003-025-08679-4. 40813467 PMC12354853

[68] Sullivan O, Sie C, Ng KM, Cotton S, Rosete C, Hamden JE, Singh AP, Lee K, Choudhary J, Kim J, et al. Early-life gut inflammation drives sex-dependent shifts in the microbiome-endocrine-brain axis. Brain Behav Immun. 2025;125:117–139. doi: 10.1016/j.bbi.2024.12.003. 39674560

[69] Jašarević E, Morrison KE, Bale TL. Sex differences in the gut microbiome-brain axis across the lifespan. Philos Trans R Soc Lond B Biol Sci. 2016;371(1688):20150122. doi: 10.1098/rstb.2015.0122. 26833840 PMC4785905

[70] Gryaznova M, Dvoretskaya Y, Burakova I, Syromyatnikov M, Popov E, Kokina A, Mikhaylov E. Dynamics of changes in the gut microbiota of healthy mice fed with lactic acid bacteria and bifidobacteria. Microorganisms. 2022;10(5):1020. doi: 10.3390/microorganisms10051020. 35630460 PMC9144108

[71] Xu X, Ocansey DKW, Pei B, Zhang Y, Wang N, Mao F. Resveratrol alleviates DSS-induced IBD in mice by regulating the intestinal microbiota-macrophage-arginine metabolism axis. Eur J Med Res. 2023;28(1):319. doi: 10.1186/s40001-023-01257-6. 37660064 PMC10474707

[72] Wang JL, Han X, Li J, Shi R, Liu L, Liao Y, Jiang H, Zhang Y, Hu J. Differential analysis of intestinal microbiota and metabolites in mice with dextran sulfate sodium-induced colitis. World J Gastroenterol. 2022;28(43):6109–6130. doi: 10.3748/wjg.v28.i43.6109. 36483152 PMC9724481

[73] Li H, Wen J, Zhang X, Dai Z, Liu M, Lei R, Luo P. Large-scale genetic correlation studies explore the causal relationship and potential mechanism between gut microbiota and COVID-19-associated risks. BMC Microbiol. 2024;24(1):292. doi: 10.1186/s12866-024-03423-0. 39103761 PMC11299294

[74] Zheng P, Zeng B, Liu M, Chen J, Pan J, Han Y, Cheng K, Zhou C, Wang H, Gui S, et al. The gut microbiome from patients with schizophrenia modulates the glutamate-glutamine-gaba cycle and schizophrenia-relevant behaviors in mice. Sci Adv. 2019;5(2):eaau8317. doi: 10.1126/sciadv.aau8317. 30775438 PMC6365110

[75] Luo C, Tian B, Zhou Y, Shang Q, Yu S, Dai M, Li Y, Chen H, Fröde TS. Deciphering the interplay among inflammatory bowel disease, gut microbiota, and inflammatory biomarkers in the risk of colorectal cancer. Mediators Inflamm. 2025;2025:4967641. doi: 10.1155/mi/4967641. 40224486 PMC11986182

[76] Uddin MK, Mahmud MR, Hasan S, Peltoniemi O, Oliviero C. Dietary micro-fibrillated cellulose improves growth, reduces diarrhea, modulates gut microbiota, and increases butyrate production in post-weaning piglets. Sci Rep. 2023;13(1):6194. doi: 10.1038/s41598-023-33291-z. 37062780 PMC10106463

[77] Zhou L, Xiao X, Zhang Q, Zheng J, Li M, Yu M, Wang X, Deng M, Zhai X. Improved glucose and lipid metabolism in the early life of female offspring by maternal dietary genistein is associated with alterations in the gut microbiota. Front Endocrinol (Lausanne). 2018;9:516. doi: 10.3389/fendo.2018.00516. 30233500 PMC6131301

[78] Sun L, Jia H, Li J, Yu M, Yang Y, Tian D, Zhang H, Zou Z. Cecal gut microbiota and metabolites might contribute to the severity of acute myocardial ischemia by impacting the intestinal permeability, oxidative stress, and energy metabolism. Front Microbiol. 2019;10:1745. doi: 10.3389/fmicb.2019.01745. 31428065 PMC6687875

[79] Cao F, Pan F, Gong X, Wang W, Xu Y. Causal relationship between gut microbiota with subcutaneous and visceral adipose tissue: a bidirectional two-sample mendelian randomization study. Front Microbiol. 2023;14:1285982. doi: 10.3389/fmicb.2023.1285982. 38029216 PMC10644100

[80] Migale bioinformatics core facility dataverse. 2022. Available from: https://entrepot.recherche.data.gouv.fr/dataverse/migale.

