# Supplementary material for: Sex modulates the long-term effects of delivery mode on microbiota–gut barrier crosstalk and colitis susceptibility in mice
Source: Gut Microbes. 2026 Apr 27;18(1):2658276. doi: 10.1080/19490976.2026.2658276 (PMC13134407; doi:10.1080/19490976.2026.2658276)
Supplement: Supl._Figures_Legend.docx — Supplemental Material [file KGMI_A_2658276_SM8410.docx]

**Supl. Fig. 1. Cesarean delivery (CSD) predisposes exacerbated colitis in post-weaning period with impaired barrier function in a sex-dependent manner.** (a) Percentage of body weight loss during DNBS-induced colitis. (b) Macroscopical assessment of colonic tissue showing damage scores. (c) Quantification of colonic goblets cells Ab+. Data are represented as scatter dot plots with individual animals for normal data. (d) Intestinal barrier integrity assessed by FITC-dextran translocation. Mean values are indicated by black dotted lines and error bars denote standard deviation (SD). Box plots and violin plots were used to non-normal distributed data. Statistical analyses were performed using Kruskal–Wallis tests followed by Dunn’s post hoc test for non-normal data and 3-way ANOVA for normal distributed data followed by Fischer’ post hoc test. N= 10. * p< 0.05, **p<0.01, ***p<0.001.
