# Supplementary material for: Sex modulates the long-term effects of delivery mode on microbiota–gut barrier crosstalk and colitis susceptibility in mice
Source: Gut Microbes. 2026 Apr 27;18(1):2658276. doi: 10.1080/19490976.2026.2658276 (PMC13134407; doi:10.1080/19490976.2026.2658276)
Supplement: Supl_tables_REVISED.docx — Supplemental Material [file KGMI_A_2658276_SM8409.docx]

**Supl. Table 1.** Animals per group, sample size, and atrition during first days of life

| AGE | Samples and animals | Groups | | | | Atrition |
| --- | --- | --- | --- | --- | --- | --- |
|  |  | VD | | CSD | |  |
|  |  | Female | Male | Female | Male |  |
| First days of life | Colonic cytokines | 5 | 5 | 5 | 5 | One batch with colon tissue removal |
|  | Lipocalin 2 (pg/ml) | 13 | 8 | 10 | 9 | Technical limitations: samples lost |
|  | sCD14 (pg/mL) | 13 | 8 | 10 | 9 | Technical limitations: samples lost |
|  | CD3^+^CD4^+^ROR-γ^+^ (%) | 15 | 10 | 10 | 10 | No exclusion |
|  | Netmoss score | 15 | 10 | 10 | 10 | No exclusion |
|  | Linda analysis | 15 | 10 | 10 | 10 | No exclusion |
|  | **Animals** | 15 | 10 | 10 | 10 | No exclusion |
| Weaning | Ileum conductance | 4 | 4 | 4 | 4 | One batch with ileum tissue removal used in electric measures in Ussing experiments. Technical limitations: samples lost |
|  | Colon conductance | 5 | 4 | 4 | 5 | One batch with colon tissue removal used in electric measures in Ussing experiments. Technical limitations: samples lost |
|  | TEER | 5 | 4 | 5 | 4 | One batch with colon tissue removal used in electric measures in Ussing experiments. Technical limitations: samples lost |
|  | TRITC COLON | 9 | 10 | 10 | 10 | Technical limitations: one sample lost |
|  | TRITC ILEUM | 10 | 10 | 10 | 10 | No exclusion |
|  | Netmoss score | 10 | 10 | 10 | 10 | No exclusion |
|  | Linda analysis | 10 | 10 | 10 | 10 | No exclusion |
|  | **Animals** | 10 | 10 | 10 | 10 | No exclusion |

**Supl. Table 2.** Animals per group, sample size and atrition post-weaning

| AGE | Samples and animals | EtOH | | | | DNBS | | | | Atrition |
| --- | --- | --- | --- | --- | --- | --- | --- | --- | --- | --- |
|  |  | VD | | CSD | | VD | | CSD | |  |
|  |  | Female | Male | Female | Male | Female | Male | Female | Male |  |
| Post-Weaning colitis | Body weight loss | 10 | 10 | 10 | 10 | 10 | 10 | 10 | 10 | No exclusion |
|  | Colonic Macroscopic score | 10 | 10 | 10 | 10 | 10 | 10 | 10 | 10 | No exclusion |
|  | AB+ goblet cells | 10 | 10 | 10 | 10 | 10 | 10 | 10 | 10 | No exclusion |
|  | FITC | 10 | 10 | 10 | 10 | 10 | 10 | 10 | 10 | No exclusion |
|  | **Animals** | 10 | 10 | 10 | 10 | 10 | 10 | 10 | 10 | No exclusion |
| Young adult | Netmoss score | 10 | 10 | 10 | 10 | 10 | 10 | 10 | 10 | No exclusion |
|  | Linda analysis | 10 | 10 | 10 | 10 | 10 | 10 | 10 | 10 | No exclusion |
|  | **Animals** | 10 | 10 | 10 | 10 | 10 | 10 | 10 | 10 | No exclusion |
| Colitis remission in adulthood | Linda analysis | 10 | 10 | 10 | 10 | 10 | 10 | 10 | 10 | No exclusion |
|  | **Animals** | 10 | 10 | 10 | 10 | 10 | 10 | 10 | 10 | No exclusion |
| Colitis reoccurrence in adulthood | Body weight loss | 10 | 10 | 10 | 10 | 9 | 7 | 10 | 9 | Technical limitations: samples lost |
|  | Colonic Macroscopic score | 10 | 10 | 10 | 10 | 9 | 7 | 10 | 9 | Technical limitations: samples lost |
|  | AB+ goblet cells | 27 | 30 | 27 | 30 | 27 | 18 | 27 | 24 | No exclusion |
|  | FITC | 9 | 10 | 10 | 9 | 9 | 6 | 9 | 8 | Technical limitations: sample lost |
|  | Colonic MPO | 9 | 10 | 10 | 10 | 10 | 10 | 10 | 10 | Technical limitations: one sample lost |
|  | Colonic IL-1aplha | 10 | 10 | 10 | 10 | 10 | 10 | 10 | 10 | No exclusion |
|  | Linda analysis | 10 | 10 | 10 | 10 | 10 | 10 | 10 | 10 | No exclusion |
|  | **Animals** | 10 | 10 | 10 | 10 | 10 | 10 | 10 | 10 | No exclusion |

**Supl. Table 3.** Linda’s significant genus (delivery effect corrected by sex): First days of life

|  | baseMean | log2FoldChange | lfcSE | stat | pvalue | padj | reject | df | Kingdom | Phylum | Class | Order | Family | Genus |
| --- | --- | --- | --- | --- | --- | --- | --- | --- | --- | --- | --- | --- | --- | --- |
| ASV27 | 42545,56467 | -4,096622554 | 1,120254669 | -3,656867198 | 0,0007356501783 | 0,005620367362 | TRUE | 40 | Bacteria | Firmicutes | Clostridia | Lachnospirales | Lachnospiraceae | Lachnospiraceae NK4A136 group |
| ASV6 | 2956,883333 | -3,97956648 | 1,117216167 | -3,562038035 | 0,0009685760377 | 0,006607072257 | TRUE | 40 | Bacteria | Bacteroidota | Bacteroidia | Bacteroidales | Prevotellaceae | Alloprevotella |
| ASV92 | 1411,797417 | -3,747010897 | 0,7634195767 | -4,908193359 | 0,00001585703343 | 0,0003371651136 | TRUE | 40 | Bacteria | Firmicutes | Clostridia | Oscillospirales | Oscillospiraceae | Intestinimonas |
| ASV3 | 10977,81997 | -3,713394474 | 1,299887169 | -2,856705229 | 0,006759463086 | 0,03002459185 | TRUE | 40 | Bacteria | Bacteroidota | Bacteroidia | Bacteroidales | Rikenellaceae | Alistipes |
| ASV5 | 2753,862964 | -3,711574988 | 0,8759544187 | -4,237178225 | 0,0001292911108 | 0,001452623656 | TRUE | 40 | Bacteria | Firmicutes | Bacilli | Staphylococcales | Staphylococcaceae | Staphylococcus |
| ASV7 | 8122,181618 | -3,691915261 | 1,071633328 | -3,445129193 | 0,001354073245 | 0,008918206545 | TRUE | 40 | Bacteria | Bacteroidota | Bacteroidia | Bacteroidales | Bacteroidaceae | Bacteroides |
| ASV77 | 4099,265869 | -3,439390068 | 0,9446335653 | -3,640978041 | 0,0007705030561 | 0,005660233989 | TRUE | 40 | Bacteria | Firmicutes | Clostridia | Lachnospirales | Lachnospiraceae | Roseburia |
| ASV165 | 1038,462322 | -3,277597115 | 0,6444924752 | -5,085547529 | 0,000009011291251 | 0,0003277888738 | TRUE | 40 | Bacteria | Firmicutes | Clostridia | Oscillospirales | Ruminococcaceae | [Eubacterium] siraeum group |
| ASV74 | 947,902861 | -3,199988975 | 0,7167192561 | -4,464773267 | 0,00006399468256 | 0,0008730703121 | TRUE | 40 | Bacteria | Deferribacterota | Deferribacteres | Deferribacterales | Deferribacteraceae | Mucispirillum |
| ASV231 | 1143,162884 | -3,162780785 | 0,6840329083 | -4,623726062 | 0,00003894116338 | 0,0006198135172 | TRUE | 40 | Bacteria | Firmicutes | Clostridia | Lachnospirales | Lachnospiraceae | Lachnospiraceae UCG-001 |
| ASV142 | 2639,865828 | -3,12354692 | 0,8684612376 | -3,596645175 | 0,0008763582974 | 0,006199423511 | TRUE | 40 | Bacteria | Firmicutes | Clostridia | Oscillospirales | Oscillospiraceae | Colidextribacter |
| ASV164 | 698,5788178 | -3,122767325 | 0,6251230196 | -4,99544446 | 0,0000120132048 | 0,0003277888738 | TRUE | 40 | Bacteria | Firmicutes | Clostridia | Oscillospirales | Ruminococcaceae | Incertae Sedis |
| ASV36 | 2383,162625 | -3,089682739 | 0,9426231692 | -3,277749625 | 0,002169345163 | 0,01183842646 | TRUE | 40 | Bacteria | Bacteroidota | Bacteroidia | Bacteroidales | Muribaculaceae | Muribaculum |
| ASV256 | 1214,205776 | -3,043596087 | 0,7281683151 | -4,179797478 | 0,0001541155673 | 0,001549267019 | TRUE | 40 | Bacteria | Firmicutes | Clostridia | Lachnospirales | Lachnospiraceae | GCA-900066575 |
| ASV130 | 1479,791272 | -2,976400255 | 0,7554057268 | -3,940134618 | 0,0003183107422 | 0,002895111988 | TRUE | 40 | Bacteria | Firmicutes | Clostridia | Lachnospirales | Lachnospiraceae | Lachnospiraceae UCG-006 |
| ASV196 | 906,2382218 | -2,956327171 | 0,6065001811 | -4,874404433 | 0,00001765262375 | 0,0003371651136 | TRUE | 40 | Bacteria | Firmicutes | Clostridia | Oscillospirales | Ruminococcaceae | Ruminococcus |
| ASV186 | 772,7148562 | -2,899308836 | 0,6449849184 | -4,495157567 | 0,0000582168807 | 0,0008553403242 | TRUE | 40 | Bacteria | Firmicutes | Clostridia | Oscillospirales | Oscillospiraceae | UCG-003 |
| ASV200 | 737,9519418 | -2,796170195 | 0,6375698827 | -4,38566857 | 0,00008180759844 | 0,00104168342 | TRUE | 40 | Bacteria | Firmicutes | Clostridia | Lachnospirales | Lachnospiraceae | ASF356 |
| ASV76 | 838,4060195 | -2,735236216 | 0,639777815 | -4,275290815 | 0,0001150099413 | 0,001372931174 | TRUE | 40 | Bacteria | Firmicutes | Clostridia | Oscillospirales | Ruminococcaceae | Anaerotruncus |
| ASV87 | 479,2886194 | -2,704287878 | 0,5201313093 | -5,199240711 | 0,000006262505713 | 0,0002990346478 | TRUE | 40 | Bacteria | Firmicutes | Bacilli | Acholeplasmatales | Acholeplasmataceae | Anaeroplasma |
| ASV260 | 789,6903249 | -2,698687487 | 0,5650802484 | -4,775759716 | 0,00002412489738 | 0,0004188959454 | TRUE | 40 | Bacteria | Firmicutes | Clostridia | Oscillospirales | Butyricicoccaceae | Butyricicoccus |
| ASV4 | 2432,133912 | -2,635745165 | 0,8822840308 | -2,987411166 | 0,004788372258 | 0,02286447753 | TRUE | 40 | Bacteria | Bacteroidota | Bacteroidia | Bacteroidales | Prevotellaceae | Prevotellaceae UCG-001 |
| ASV55 | 815,0131524 | -2,55789524 | 0,6098020409 | -4,194632139 | 0,0001472842444 | 0,001549267019 | TRUE | 40 | Bacteria | Proteobacteria | Gammaproteobacteria | Burkholderiales | Sutterellaceae | Parasutterella |
| ASV129 | 1624,750624 | -2,496631902 | 0,9284361095 | -2,689072384 | 0,01039732237 | 0,04513383119 | TRUE | 40 | Bacteria | Firmicutes | Clostridia | Lachnospirales | Lachnospiraceae | Lachnoclostridium |
| ASV286 | 1066,801987 | -2,444141195 | 0,7455887301 | -3,278135916 | 0,002167012646 | 0,01183842646 | TRUE | 40 | Bacteria | Firmicutes | Clostridia | Lachnospirales | Lachnospiraceae | Lachnospiraceae FCS020 group |
| ASV115 | 1110,580246 | -2,409816769 | 0,7074023905 | -3,406571424 | 0,001510720742 | 0,00961825539 | TRUE | 40 | Bacteria | Firmicutes | Clostridia | Lachnospirales | Lachnospiraceae | A2 |
| ASV131 | 1317,446281 | -2,341162987 | 0,7885462016 | -2,968961086 | 0,00502947131 | 0,0234299761 | TRUE | 40 | Bacteria | Firmicutes | Clostridia | Oscillospirales | Oscillospiraceae | Oscillibacter |
| ASV31 | 749,9391046 | -2,291386635 | 0,7184226997 | -3,18946859 | 0,002769794366 | 0,01469529789 | TRUE | 40 | Bacteria | Bacteroidota | Bacteroidia | Bacteroidales | Rikenellaceae | Rikenellaceae RC9 gut group |
| ASV244 | 365,4209876 | -2,282812395 | 0,4191442606 | -5,446364438 | 0,000002829748741 | 0,0002990346478 | TRUE | 40 | Bacteria | Actinobacteriota | Coriobacteriia | Coriobacteriales | Atopobiaceae | Coriobacteriaceae UCG-002 |
| ASV149 | 712,1416977 | -2,212825674 | 0,7098928586 | -3,117126264 | 0,003376049282 | 0,01653398494 | TRUE | 40 | Bacteria | Firmicutes | Clostridia | Oscillospirales | Ruminococcaceae | UBA1819 |
| ASV207 | 251,6119714 | -1,92977605 | 0,3638016106 | -5,304473631 | 0,000004467439265 | 0,0002990346478 | TRUE | 40 | Bacteria | Firmicutes | Clostridia | Lachnospirales | Lachnospiraceae | [Eubacterium] ventriosum group |
| ASV299 | 277,9045801 | -1,910162268 | 0,3811791438 | -5,011193028 | 0,00001142507789 | 0,0003277888738 | TRUE | 40 | Bacteria | Firmicutes | Clostridia | Oscillospirales | Oscillospiraceae | NK4A214 group |
| ASV318 | 388,2352112 | -1,876738925 | 0,4723359345 | -3,973313882 | 0,0002881429948 | 0,0027517656 | TRUE | 40 | Bacteria | Firmicutes | Clostridia | Lachnospirales | Lachnospiraceae | [Eubacterium] xylanophilum group |
| ASV421 | 359,3060946 | -1,77527231 | 0,4721434871 | -3,760027107 | 0,0005436734803 | 0,004326734781 | TRUE | 40 | Bacteria | Firmicutes | Clostridia | Oscillospirales | Oscillospiraceae | UCG-005 |
| ASV126 | 496,3710804 | -1,769960951 | 0,5605897285 | -3,157319623 | 0,003025254332 | 0,01561685345 | TRUE | 40 | Bacteria | Firmicutes | Clostridia | Lachnospirales | Lachnospiraceae | Tyzzerella |
| ASV134 | 225,4287762 | -1,699827053 | 0,3221862556 | -5,275914236 | 0,000004896712735 | 0,0002990346478 | TRUE | 40 | Bacteria | Firmicutes | Clostridia | Clostridiales | Clostridiaceae | Candidatus Arthromitus |
| ASV430 | 234,0085697 | -1,656600318 | 0,3387131097 | -4,890865663 | 0,00001675406998 | 0,0003371651136 | TRUE | 40 | Bacteria | Firmicutes | Clostridia | Peptococcales | Peptococcaceae | Peptococcus |
| ASV319 | 265,5191125 | -1,629779098 | 0,4202951651 | -3,877701275 | 0,0003836020026 | 0,003185564456 | TRUE | 40 | Bacteria | Desulfobacterota | Desulfovibrionia | Desulfovibrionales | Desulfovibrionaceae | Bilophila |
| ASV308 | 214,5319995 | -1,342231868 | 0,3969637639 | -3,381245317 | 0,001622880825 | 0,009999039924 | TRUE | 40 | Bacteria | Firmicutes | Clostridia | Oscillospirales | Butyricicoccaceae | UCG-009 |
| ASV305 | 172,053493 | -1,255288045 | 0,3746489391 | -3,350571466 | 0,001769382733 | 0,01056100319 | TRUE | 40 | Bacteria | Firmicutes | Clostridia | Oscillospirales | Ruminococcaceae | Paludicola |
| ASV465 | 143,9718196 | -1,198154351 | 0,3057696312 | -3,918487085 | 0,0003396235141 | 0,0029485496 | TRUE | 40 | Bacteria | Firmicutes | Clostridia | Lachnospirales | Lachnospiraceae | Tuzzerella |
| ASV53 | 153,6821277 | -1,083457611 | 0,3727471632 | -2,906682379 | 0,005930014087 | 0,02696744502 | TRUE | 40 | Bacteria | Firmicutes | Bacilli | Lactobacillales | Carnobacteriaceae | Atopostipes |
| ASV317 | 128,1409064 | -0,9581255002 | 0,2877073487 | -3,330208646 | 0,001873513818 | 0,01084367089 | TRUE | 40 | Bacteria | Firmicutes | Clostridia | Peptostreptococcales-Tissierellales | Anaerovoracaceae | [Eubacterium] nodatum group |
| ASV30 | 34007,47566 | 1,831787036 | 0,5874644845 | 3,118123876 | 0,003366895429 | 0,01653398494 | TRUE | 40 | Bacteria | Firmicutes | Bacilli | Lactobacillales | Streptococcaceae | Streptococcus |

*Green: increased in VD and purple: increased in CSD

**Supl. table 4.** Linda’s significant species

|  | Timepoint | LinDA | baseMean | log2FoldChange | lfcSE | stat | pvalue | padj | reject | df | Kingdom | Phylum | Class | Order | Family | Genus | Species |
| --- | --- | --- | --- | --- | --- | --- | --- | --- | --- | --- | --- | --- | --- | --- | --- | --- | --- |
| **ASV31** | **First days of life** | **Mode of delivery corrected by sex** | **577.0343089** | **-2.091985404** | **0.742518487** | **-2.817418611** | **0.007486127** | **0.036045538** | **TRUE** | **40** | **Bacteria** | ***Bacteroidota*** | ***Bacteroidia*** | ***Bacteroidales*** | ***Rikenellaceae*** | ***Rikenellaceae RC9 gut group*** | ***NA*** |
| **ASV31** | **Young adult** | **Mode of delivery corrected by sex** | **24030.01934** | **-2.254330881** | **0.432621038** | **-5.210867437** | **2.35329E-06** | **0.002369766** | **TRUE** | **61** | **Bacteria** | ***Bacteroidota*** | ***Bacteroidia*** | ***Bacteroidales*** | ***Rikenellaceae*** | ***Rikenellaceae RC9 gut group*** | ***NA*** |
| **ASV31** | **Colitis reocurrrence in adulthood** | **Mode of delivery corrected by sex** | **25720.28952** | **-2.786083978** | **0.589071685** | **-4.729617886** | **1.40783E-05** | **0.007447414** | **TRUE** | **60** | **Bacteria** | ***Bacteroidota*** | ***Bacteroidia*** | ***Bacteroidales*** | ***Rikenellaceae*** | ***Rikenellaceae RC9 gut group*** | ***NA*** |
| **ASV4** | **Colitis remission in adulthood** | **Sex effect corrected by delivery mode** | **16705.05944** | **1.839591688** | **0.448294317** | **4.103535598** | **0.00012462** | **0.018960084** | **TRUE** | **60** | **Bacteria** | ***Bacteroidota*** | ***Bacteroidia*** | ***Bacteroidales*** | ***Prevotellaceae*** | ***Prevotellaceae UCG-001*** | ***NA*** |
| **ASV4** | **Colitis reocurrrence in adulthood** | **Sex effect corrected by delivery mode** | **31515.66096** | **1.338295454** | **0.357963484** | **3.738636801** | **0.000415211** | **0.036607783** | **TRUE** | **60** | **Bacteria** | ***Bacteroidota*** | ***Bacteroidia*** | ***Bacteroidales*** | ***Prevotellaceae*** | ***Prevotellaceae UCG-001*** | ***NA*** |
| **ASV12** | **Colitis reocurrrence in adulthood** | **Sex effect corrected by delivery mode** | **28213.10187** | **-2.589112752** | **0.490986185** | **-5.273290438** | **1.92886E-06** | **0.000510182** | **TRUE** | **60** | **Bacteria** | ***Firmicutes*** | ***Bacilli*** | ***Lactobacillales*** | ***Lactobacillaceae*** | ***Lactobacillus*** | ***iatae/johnsonii/taiwanensis*** |

* Purple: increased in CSD; brown: increased in females, and blue: increased in males.

**Supl. table 5.** Linda’s significant genus (delivery effect corrected by sex): Weaning

|  | **baseMean** | **log2FoldChange** | **lfcSE** | **stat** | **pvalue** | **padj** | **reject** | **df** | **Kingdom** | **Phylum** | **Class** | **Order** | **Family** | **Genus** |
| --- | --- | --- | --- | --- | --- | --- | --- | --- | --- | --- | --- | --- | --- | --- |
| ASV317 | 464,0017189 | -1,88278828 | 0,4983555831 | -3,778001781 | 0,000361283201 | 0,01661902724 | TRUE | 61 | Bacteria | Firmicutes | Clostridia | Peptostreptococcales-Tissierellales | Anaerovoracaceae | [Eubacterium] nodatum group |
| ASV465 | 198,9687166 | -1,326310763 | 0,4070482485 | -3,258362535 | 0,001833590285 | 0,04217257655 | TRUE | 61 | Bacteria | Firmicutes | Clostridia | Lachnospirales | Lachnospiraceae | Tuzzerella |
| ASV27 | 142258,6655 | -0,7562144171 | 0,224360644 | -3,370530604 | 0,001305917008 | 0,04004812158 | TRUE | 61 | Bacteria | Firmicutes | Clostridia | Lachnospirales | Lachnospiraceae | Lachnospiraceae NK4A136 group |
| ASV9 | 34509,12736 | 1,425727851 | 0,3296391772 | 4,32511652 | 0,00005741668993 | 0,005282335473 | TRUE | 61 | Bacteria | Bacteroidota | Bacteroidia | Bacteroidales | Marinifilaceae | Odoribacter |

*Green: increased in VD and purple: increased in CSD.

**Supl. table 6.** Linda’s significant genus (delivery effect corrected by sex): Young adult

|  | **baseMean** | **log2FoldChange** | **lfcSE** | **stat** | **pvalue** | **padj** | **reject** | **df** | **Kingdom** | **Phylum** | **Class** | **Order** | **Family** | **Genus** |
| --- | --- | --- | --- | --- | --- | --- | --- | --- | --- | --- | --- | --- | --- | --- |
| ASV31 | 37480,49792 | -2,389571208 | 0,4206157164 | -5,681126774 | 0,0000003985085344 | 0,00003387322542 | TRUE | 61 | Bacteria | Bacteroidota | Bacteroidia | Bacteroidales | Rikenellaceae | Rikenellaceae RC9 gut group |
| ASV55 | 17609,68164 | -1,537822852 | 0,4011776295 | -3,833271695 | 0,0003017053018 | 0,005128990131 | TRUE | 61 | Bacteria | Proteobacteria | Gammaproteobacteria | Burkholderiales | Sutterellaceae | Parasutterella |
| ASV12 | 61125,86102 | -1,427765142 | 0,4202826962 | -3,397154237 | 0,001203707799 | 0,01461645184 | TRUE | 61 | Bacteria | Firmicutes | Bacilli | Lactobacillales | Lactobacillaceae | Lactobacillus |
| ASV27 | 115694,1705 | 0,6923687106 | 0,1704971863 | 4,060880567 | 0,0001416853859 | 0,003010814451 | TRUE | 61 | Bacteria | Firmicutes | Clostridia | Lachnospirales | Lachnospiraceae | Lachnospiraceae NK4A136 group |
| ASV74 | 533,2964788 | 1,74064189 | 0,5057644158 | 3,441606083 | 0,001049735215 | 0,01461645184 | TRUE | 61 | Bacteria | Deferribacterota | Deferribacteres | Deferribacterales | Deferribacteraceae | Mucispirillum |
| ASV260 | 895,4574962 | 1,816342615 | 0,4316989349 | 4,207428993 | 0,00008613348755 | 0,003010814451 | TRUE | 61 | Bacteria | Firmicutes | Clostridia | Oscillospirales | Butyricicoccaceae | Butyricicoccus |
| ASV294 | 155,603427 | 1,852969301 | 0,5595668774 | 3,311434925 | 0,00156285455 | 0,01660532959 | TRUE | 61 | Bacteria | Firmicutes | Clostridia | Lachnospirales | Lachnospiraceae | Acetatifactor |
| ASV318 | 87,73516491 | 1,861579479 | 0,4582085314 | 4,062734217 | 0,0001408036069 | 0,003010814451 | TRUE | 61 | Bacteria | Firmicutes | Clostridia | Lachnospirales | Lachnospiraceae | [Eubacterium] xylanophilum group |

*Green: increased in VD and purple: increased in CSD

**Supl. table 7.** Linda’s significant genus (delivery effect corrected by sex): Colitis remission in adulthood

|  | baseMean | log2FoldChange | lfcSE | stat | pvalue | padj | reject | df | Kingdom | Phylum | Class | Order | Family | Genus |
| --- | --- | --- | --- | --- | --- | --- | --- | --- | --- | --- | --- | --- | --- | --- |
| ASV317 | 842,1603121 | -1,676426813 | 0,4670314754 | -3,589537111 | 0,0006677866072 | 0,03205375715 | TRUE | 60 | Bacteria | Firmicutes | Clostridia | Peptostreptococcales-Tissierellales | Anaerovoracaceae | [Eubacterium] nodatum group |
| ASV31 | 26069,86268 | -1,550848821 | 0,4207524031 | -3,68589415 | 0,0004917814799 | 0,03205375715 | TRUE | 60 | Bacteria | Bacteroidota | Bacteroidia | Bacteroidales | Rikenellaceae | Rikenellaceae RC9 gut group |

*Green: increased in VD and purple: increased in CSD

**Supl. table 8.** Linda’s significant genus (delivery effect corrected by sex): Colitis reocurrance in adulthood

|  | **baseMean** | **log2FoldChange** | **lfcSE** | **stat** | **pvalue** | **padj** | **reject** | **df** | **Kingdom** | **Phylum** | **Class** | **Order** | **Family** | **Genus** |
| --- | --- | --- | --- | --- | --- | --- | --- | --- | --- | --- | --- | --- | --- | --- |
| ASV292 | 957,3922994 | -2,892748368 | 0,7724406632 | -3,744945735 | 0,0004068558959 | 0,01708794763 | TRUE | 60 | Bacteria | Bacteroidota | Bacteroidia | Bacteroidales | Tannerellaceae | Parabacteroides |
| ASV31 | 29736,47578 | -2,691796724 | 0,5644268836 | -4,769079578 | 0,00001221888537 | 0,001026386371 | TRUE | 60 | Bacteria | Bacteroidota | Bacteroidia | Bacteroidales | Rikenellaceae | Rikenellaceae RC9 gut group |
| ASV106 | 1398,982313 | -2,52863327 | 0,7124296482 | -3,549309431 | 0,0007577958372 | 0,01950948256 | TRUE | 60 | Bacteria | Firmicutes | Bacilli | Erysipelotrichales | Erysipelotrichaceae | Turicibacter |
| ASV244 | 493,7863506 | -2,209625284 | 0,6733821827 | -3,281383649 | 0,00172412988 | 0,01950948256 | TRUE | 60 | Bacteria | Actinobacteriota | Coriobacteriia | Coriobacteriales | Atopobiaceae | Coriobacteriaceae UCG-002 |
| ASV54 | 6196,768388 | -2,193101222 | 0,7079184346 | -3,097957497 | 0,00296327241 | 0,02765720916 | TRUE | 60 | Bacteria | Firmicutes | Bacilli | Lactobacillales | Lactobacillaceae | HT002 |
| ASV465 | 809,7650199 | -1,624674825 | 0,4874129228 | -3,333261694 | 0,001474548064 | 0,01950948256 | TRUE | 60 | Bacteria | Firmicutes | Clostridia | Lachnospirales | Lachnospiraceae | Tuzzerella |
| ASV12 | 32614,31074 | -1,439848585 | 0,4905781285 | -2,935003625 | 0,004721104214 | 0,0396572754 | TRUE | 60 | Bacteria | Firmicutes | Bacilli | Lactobacillales | Lactobacillaceae | Lactobacillus |
| ASV260 | 1373,351684 | 1,498014283 | 0,4461111133 | 3,357939847 | 0,001368184053 | 0,01950948256 | TRUE | 60 | Bacteria | Firmicutes | Clostridia | Oscillospirales | Butyricicoccaceae | Butyricicoccus |
| ASV421 | 228,9463478 | 1,591347133 | 0,4886825592 | 3,256402551 | 0,001858045958 | 0,01950948256 | TRUE | 60 | Bacteria | Firmicutes | Clostridia | Oscillospirales | Oscillospiraceae | UCG-005 |
| ASV286 | 184,0879623 | 2,077376341 | 0,6074863076 | 3,419626607 | 0,001133150922 | 0,01950948256 | TRUE | 60 | Bacteria | Firmicutes | Clostridia | Lachnospirales | Lachnospiraceae | Lachnospiraceae FCS020 group |

*Green: increased in VD and purple: increased in CSD

**Supl. table 9.** Linda’s significant genus (sex effect corrected by delivery): young adult

|  | **baseMean** | **log2FoldChange** | **lfcSE** | **stat** | **pvalue** | **padj** | **reject** | **df** | **Kingdom** | **Phylum** | **Class** | **Order** | **Family** | **Genus** |
| --- | --- | --- | --- | --- | --- | --- | --- | --- | --- | --- | --- | --- | --- | --- |
| ASV213 | 368,84558 | -2,245767373 | 0,6572622781 | -3,416851153 | 0,001133013941 | 0,03210206167 | TRUE | 61 | Bacteria | Firmicutes | Bacilli | Lactobacillales | Lactobacillaceae | Limosilactobacillus |
| ASV31 | 37480,49792 | -1,605871021 | 0,4204102875 | -3,819770992 | 0,0003153220042 | 0,01340118518 | TRUE | 61 | Bacteria | Bacteroidota | Bacteroidia | Bacteroidales | Rikenellaceae | Rikenellaceae RC9 gut group |
| ASV149 | 903,445318 | 1,805344113 | 0,4026023913 | 4,484186263 | 0,00003292829424 | 0,002798905011 | TRUE | 61 | Bacteria | Firmicutes | Clostridia | Oscillospirales | Ruminococcaceae | UBA1819 |

*Brown: increased in females and blue: increased in males

**Supl. table 10.** Linda’s significant genus (sex effect corrected by delivery): Colitis remission in adulthood

|  | **baseMean** | **log2FoldChange** | **lfcSE** | **stat** | **pvalue** | **padj** | **reject** | **df** | **Kingdom** | **Phylum** | **Class** | **Order** | **Family** | **Genus** |
| --- | --- | --- | --- | --- | --- | --- | --- | --- | --- | --- | --- | --- | --- | --- |
| ASV213 | 654,9130271 | -3,089999824 | 0,6544064772 | -4,721835634 | 0,00001447633707 | 0,0005513688713 | TRUE | 60 | Bacteria | Firmicutes | Bacilli | Lactobacillales | Lactobacillaceae | Limosilactobacillus |
| ASV29 | 343,8119057 | -2,192890636 | 0,5773555856 | -3,798163023 | 0,0003425058601 | 0,004697223224 | TRUE | 60 | Bacteria | Proteobacteria | Gammaproteobacteria | Enterobacterales | Enterobacteriaceae | Escherichia-Shigella |
| ASV131 | 6103,72532 | -1,794631214 | 0,4472484213 | -4,012604915 | 0,0001690662439 | 0,002705059902 | TRUE | 60 | Bacteria | Firmicutes | Clostridia | Oscillospirales | Oscillospiraceae | Oscillibacter |
| ASV115 | 2835,859796 | -1,753262546 | 0,601819921 | -2,913267715 | 0,005018021982 | 0,03705616233 | TRUE | 60 | Bacteria | Firmicutes | Clostridia | Lachnospirales | Lachnospiraceae | A2 |
| ASV286 | 1217,804903 | -1,730123044 | 0,6016135224 | -2,875804781 | 0,005570712268 | 0,03819916984 | TRUE | 60 | Bacteria | Firmicutes | Clostridia | Lachnospirales | Lachnospiraceae | Lachnospiraceae FCS020 group |
| ASV318 | 197,663663 | -1,665278637 | 0,5186632107 | -3,210712853 | 0,002128663657 | 0,02043517111 | TRUE | 60 | Bacteria | Firmicutes | Clostridia | Lachnospirales | Lachnospiraceae | [Eubacterium] xylanophilum group |
| ASV319 | 291,0556255 | -1,529922313 | 0,4504359937 | -3,396536543 | 0,001216254366 | 0,01297337991 | TRUE | 60 | Bacteria | Desulfobacterota | Desulfovibrionia | Desulfovibrionales | Desulfovibrionaceae | Bilophila |
| ASV31 | 26069,86268 | -1,284913715 | 0,4203413113 | -3,056834246 | 0,003337510301 | 0,0267000824 | TRUE | 60 | Bacteria | Bacteroidota | Bacteroidia | Bacteroidales | Rikenellaceae | Rikenellaceae RC9 gut group |
| ASV200 | 4363,309636 | -1,207165666 | 0,3412642516 | -3,537334077 | 0,0007867476706 | 0,009440972047 | TRUE | 60 | Bacteria | Firmicutes | Clostridia | Lachnospirales | Lachnospiraceae | ASF356 |
| ASV4 | 23385,47041 | 1,853858274 | 0,4426934482 | 4,187679491 | 0,00009370675542 | 0,001799169704 | TRUE | 60 | Bacteria | Bacteroidota | Bacteroidia | Bacteroidales | Prevotellaceae | Prevotellaceae UCG-001 |
| ASV106 | 486,1355394 | 2,193936403 | 0,7101413434 | 3,089436242 | 0,003037437948 | 0,02650854937 | TRUE | 60 | Bacteria | Firmicutes | Bacilli | Erysipelotrichales | Erysipelotrichaceae | Turicibacter |
| ASV11 | 99,67384955 | 2,449395484 | 0,4896749279 | 5,002084739 | 0,000005247854873 | 0,0005037940678 | TRUE | 60 | Bacteria | Actinobacteriota | Actinobacteria | Corynebacteriales | Corynebacteriaceae | Corynebacterium |
| ASV5 | 92,98613217 | 2,66996175 | 0,5713453477 | 4,673113662 | 0,00001723027723 | 0,0005513688713 | TRUE | 60 | Bacteria | Firmicutes | Bacilli | Staphylococcales | Staphylococcaceae | Staphylococcus |
| ASV165 | 83,82038605 | 2,807032933 | 0,666563282 | 4,21120246 | 0,00008648682303 | 0,001799169704 | TRUE | 60 | Bacteria | Firmicutes | Clostridia | Oscillospirales | Ruminococcaceae | [Eubacterium] siraeum group |

*Brown: increased in females and blue: increased in males

**Supl. table 11.** Linda’s significant genus (sex effect corrected by delivery): Colitis reocurrance in adulthood

|  | **baseMean** | **log2FoldChange** | **lfcSE** | **stat** | **pvalue** | **padj** | **reject** | **df** | **Kingdom** | **Phylum** | **Class** | **Order** | **Family** | **Genus** |
| --- | --- | --- | --- | --- | --- | --- | --- | --- | --- | --- | --- | --- | --- | --- |
| ASV54 | 6196,768388 | -3,080716867 | 0,7072267701 | -4,356052397 | 0,00005255404803 | 0,001471513345 | TRUE | 60 | Bacteria | Firmicutes | Bacilli | Lactobacillales | Lactobacillaceae | HT002 |
| ASV12 | 32614,31074 | -2,712670138 | 0,4900988141 | -5,534945321 | 0,0000007233667419 | 0,00006076280632 | TRUE | 60 | Bacteria | Firmicutes | Bacilli | Lactobacillales | Lactobacillaceae | Lactobacillus |
| ASV318 | 357,3645739 | -2,440974157 | 0,5516166601 | -4,425127691 | 0,0000413343674 | 0,001471513345 | TRUE | 60 | Bacteria | Firmicutes | Clostridia | Lachnospirales | Lachnospiraceae | [Eubacterium] xylanophilum group |
| ASV352 | 1163,195141 | -2,217063082 | 0,6092532889 | -3,638984183 | 0,0005710677125 | 0,01086064441 | TRUE | 60 | Bacteria | Firmicutes | Clostridia | Peptostreptococcales-Tissierellales | Peptostreptococcaceae | Romboutsia |
| ASV130 | 694,7244577 | -1,775049478 | 0,5934483967 | -2,991076374 | 0,004028746652 | 0,03629625474 | TRUE | 60 | Bacteria | Firmicutes | Clostridia | Lachnospirales | Lachnospiraceae | Lachnospiraceae UCG-006 |
| ASV330 | 514,2195388 | -1,73470255 | 0,4818857851 | -3,599820961 | 0,0006464669294 | 0,01086064441 | TRUE | 60 | Bacteria | Actinobacteriota | Coriobacteriia | Coriobacteriales | Eggerthellaceae | Enterorhabdus |
| ASV408 | 372,991188 | -1,599236879 | 0,5234951202 | -3,054922228 | 0,003355940766 | 0,03523737804 | TRUE | 60 | Bacteria | Firmicutes | Clostridia | Monoglobales | Monoglobaceae | Monoglobus |
| ASV319 | 788,7658603 | -1,573155175 | 0,4902808051 | -3,208681961 | 0,002141513188 | 0,02569815826 | TRUE | 60 | Bacteria | Desulfobacterota | Desulfovibrionia | Desulfovibrionales | Desulfovibrionaceae | Bilophila |
| ASV421 | 228,9463478 | -1,448208619 | 0,4882050969 | -2,966393895 | 0,004320982708 | 0,03629625474 | TRUE | 60 | Bacteria | Firmicutes | Clostridia | Oscillospirales | Oscillospiraceae | UCG-005 |
| ASV4 | 36428,05276 | 1,214967094 | 0,3679874357 | 3,30165374 | 0,001622209037 | 0,02271092651 | TRUE | 60 | Bacteria | Bacteroidota | Bacteroidia | Bacteroidales | Prevotellaceae | Prevotellaceae UCG-001 |

*Brown: increased in females and blue: increased in males
