# Supplementary figures and images for: Sex modulates the long-term effects of delivery mode on microbiota–gut barrier crosstalk and colitis susceptibility in mice
Source: Gut Microbes. 2026 Apr 27;18(1):2658276. doi: 10.1080/19490976.2026.2658276 (PMC13134407; doi:10.1080/19490976.2026.2658276)

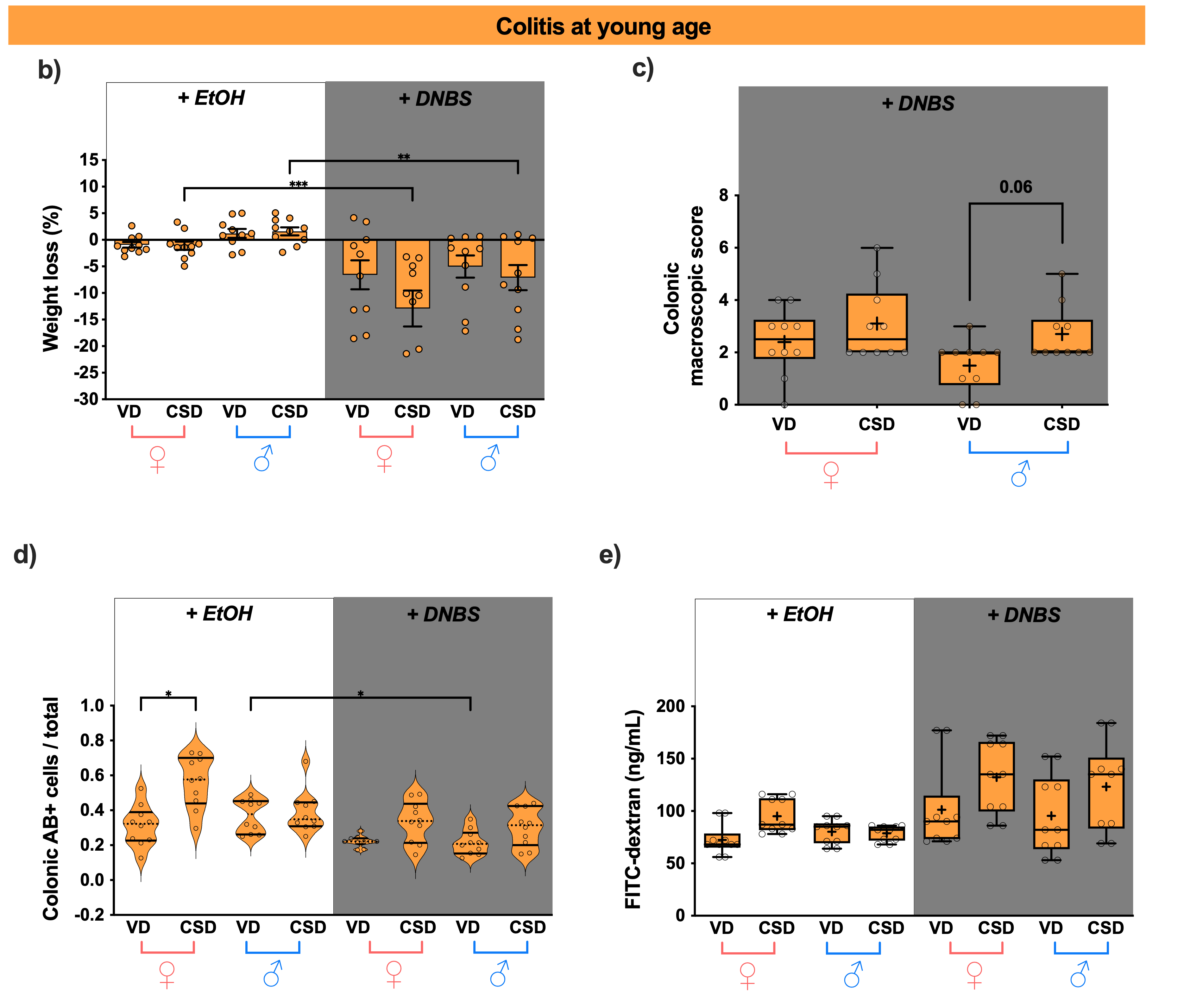

Supplement: Sup. Figures — Sup. Fig. 1 [file KGMI_A_2658276_SM8404.tif]
